# Track and dive-based movement metrics do not predict the number of prey encountered by a marine predator

**DOI:** 10.1186/s40462-022-00361-2

**Published:** 2023-01-21

**Authors:** Hassen Allegue, Denis Réale, Baptiste Picard, Christophe Guinet

**Affiliations:** 1 Département des Sciences Biologiques, Université du Québec à Montréal, Montréal, QC Canada; 2 Centre d’Etudes Biologiques de Chizé, UMR7372 CNRS-La Rochelle Université, Villiers en Bois, France

**Keywords:** Accelerometry, Area-restricted search, Diving behavior, Foraging behavior, Marine predator, Prey encounter events

## Abstract

**Background:**

Studying animal movement in the context of the optimal foraging theory has led to the development of simple movement metrics for inferring feeding activity. Yet, the predictive capacity of these metrics in natural environments has been given little attention, raising serious questions of the validity of these metrics. The aim of this study is to test whether simple continuous movement metrics predict feeding intensity in a marine predator, the southern elephant seal (SES; *Mirounga leonine*), and investigate potential factors influencing the predictive capacity of these metrics.

**Methods:**

We equipped 21 female SES from the Kerguelen Archipelago with loggers and recorded their movements during post-breeding foraging trips at sea. From accelerometry, we estimated the number of prey encounter events (nPEE) and used it as a reference for feeding intensity. We also extracted several track- and dive-based movement metrics and evaluated how well they explain and predict the variance in nPEE. We conducted our analysis at two temporal scales (dive and day), with two dive profile resolutions (high at 1 Hz and low with five dive segments), and two types of models (linear models and regression trees).

**Results:**

We found that none of the movement metrics predict nPEE with satisfactory power. The vertical transit rates (primarily the ascent rate) during dives had the best predictive performance among all metrics. Dive metrics performed better than track metrics and all metrics performed on average better at the scale of days than the scale of dives. However, the performance of the models at the scale of days showed higher variability among individuals suggesting distinct foraging tactics. Dive-based metrics performed better when computed from high-resolution dive profiles than low-resolution dive profiles. Finally, regression trees produced more accurate predictions than linear models.

**Conclusions:**

Our study reveals that simple movement metrics do not predict feeding activity in free-ranging marine predators. This could emerge from differences between individuals, temporal scales, and the data resolution used, among many other factors. We conclude that these simple metrics should be avoided or carefully tested a priori with the studied species and the ecological context to account for significant influencing factors.

**Supplementary Information:**

The online version contains supplementary material available at 10.1186/s40462-022-00361-2.

## Background

Foraging has a central role in the evolution of species as it directly affects the fitness of individuals via the probability of survival and reproduction [1]. A key question behavioral ecologists have been interested in is how organisms adopt a hierarchical decision-making process to improve foraging efficiency [2]. For instance, foragers can increase their energy intake rate while minimizing some costs, such as the time searching, capturing, and handling prey, or the risk of predation. In conservation, understanding the spatiotemporal variation of foraging behavior in response to resource distribution is fundamental for the protection and management of endangered species [3, 4]. And yet, direct observations of the interaction between predators and their prey in free-ranging species are often challenging or impossible for a variety of reasons such as remoteness and large home ranges. The development of bio-logging technologies in the last decades has helped address some of these challenges [5].

Recent developments in bio-logging devices allow researchers to collect accurate empirical observations of feeding behavior [6, 7]. Feeding events can be directly observed using animal-borne cameras [8], or indirectly from physiological measurements such as changes in stomach temperature [9]. Feeding attempts can also be inferred from high-resolution data of animal movement measured by accelerometers [10, 11]. Although these technologies have considerably contributed to our understanding of the foraging behavior of captive and free-ranging animals, they come with several logistic and methodological limitations [7]. For example, accelerometers deployed on harbor seals (*Phoca vitulina*), with 16 Hz sampling frequency, were limited to a recording period between one and two months due to the high battery consumption of the devices [12]. In addition, high-resolution data requires physical recovery as the data is generally too large to be transferred through satellite communications [12–14]. Consequently, researchers often rely on lower-resolution data, such as summarized dive profiles [15], from which they use movement proxies to infer feeding behavior [reviewed in 7, 16].

Movement metrics inferring feeding behavior have been developed based on the optimal foraging theory, which posits that foraging animals improve their fitness when adjusting their behavior in a way that maximizes their net rate energy intake in response to environmental constraints [17]. One aspect of the optimal foraging theory focuses on movement patterns that animals adopt while foraging [17]. Animals should adopt an area-restricted search (ARS) to maximize resource encounter rate and minimize costs of movement [18]. The ARS has two distinct search modes. First, an intensive search mode, triggered by resource encounters or environmental cues, that is characterized by slow speeds and large turning angles (i.e., tortuous movement). In the intensive search mode, foragers remain in the same area and thus increase the probability of encountering and consuming additional food items. Second, foragers switch to an extensive search mode after repeated unsuccessful resource encounters for which they increase speed and move in a relatively straight line to find another resource patch [19]. Therefore, movement metrics infer feeding intensity by quantifying search intensity along the track assuming a high correlation between feeding and search behavior [7].

Along with the ARS hypothesis, several track-based metrics (i.e., on the horizontal dimension of the animal movement) have been used to quantify foraging search intensity from which feeding activity can be inferred [7]. For example, transit rate and turning angle are assumed to correlate with resource encounter [13, 20–23]. The move persistence metric captures the autocorrelation in both transit rate and turning angle along the track’s trajectory [24, 25]. Low movement correlation (high persistence) represents high variation in speed and turning angle over time, which reflects an ARS behavior. In contrast, high correlation in movement results from constant and directional movements, which represents a transiting behavior [26]. Other metrics rely on the time the forager resides in an area, where higher residence time values should reveal higher search intensity due to higher prey density [27–29].

Air-breathing diving marine predators such as marine mammals, seabirds, and sea turtles face additional challenges when foraging due to physiological constraints (e.g., related to oxygen stores, [30]) and to searching for heterogenous and difficult to predict resource patches [31]. The feeding behavior of these species can be inferred from metrics associated with the vertical dimension of their movements, using data of diving profiles in addition to the horizontal dimension [7]. As diving predators must repeatedly return to the surface to breathe, they have been studied under the framework of the central place foraging theory, where foragers travel back and forth from a home base (e.g., a nest) to a distant foraging location [32]. In the context of diving predators, the surface is acting as the central place and oxygen is the resource to maximize [33]. This modification of the original central place foraging theory led to the development of the optimal diving theory [30].

The optimal diving theory posits that predators adjust their diving behavior to maximize the time at the bottom phase of the dive, where prey capture is assumed to occur [34–36]. Feeding behavior can be inferred from dive-based metrics that reflect the improvement of dive efficiency during more successful dives—i.e., the ratio between the duration of the dive bottom phase and the total dive duration, which includes the dive duration and the post-dive surface duration [35–37]. For example, Brünnich’s guillemots (*Uria lomvia*) increase bottom duration [38, 39], penguins shorten transit duration by increasing swimming speed or reducing body angle [40–43], and southern elephant seals shorten the recovery surface time [44, 45] in response to an increase of feeding activity during dives.

The ARS hypothesis has also been used to infer feeding behavior on the vertical dimension by identifying prey patch exploitation periods from dive profiles [46–48]. Diving predators are thus assumed to decrease their vertical speed and increase vertical sinuosity when encountering prey patches [13]. In several free-ranging penguin species, wiggles (or undulations) in the bottom phase of the dive correlate with feeding events as measured from drops in esophageal temperatures [49], beak openings [50], and video records [51]. Additionally, Heerah et al. [48] found that 77% of prey capture attempts in southern elephant seals, inferred from accelerometry, occurred during dive segments with high vertical sinuosity. The cumulative time of these high vertical sinuosity dive segments were defined as the hunting time [48].

Although several track- and dive-based metrics have been validated in (semi-)controlled experimental setups [52–54], they have rarely been tested on free-ranging species in natural conditions, which raises questions on their reliability as general proxies for feeding activity [45, 55–57]. The theoretical models developed from the optimal foraging and diving theory do not account for many ecological and physiological factors that may modulate predator movements. For example, optimal diving theory models assume that prey patches are uniformly distributed and have the same quality [30, 34]. Since this is typically not the case, maximizing time at the bottom of the dive may not always represent the most efficient foraging tactic [36]. Thums et al. [58] found that southern elephant seals reduce the dive bottom duration but increase descent and ascent rates in regions of higher quality which were inferred from changes in the seal body condition. The accuracy in the relationship between movement metrics and direct observations of feeding attempts may vary between species, habitats, and temporal scales [8, 39, 44, 56, 59–61]. Watanabe et al. [61] found that Adélie penguins (*Pygoscelis adeliae*) increase dive duration at the scale of dives but decrease it at the scale of bouts as the krill density increases.

Many studies that investigated the relationship between movement metrics and feeding behavior in free-ranging species did not explicitly test the capacity of the metrics to predict feeding intensity [13, 21, 44, 48, 58, 61]. For the studies that did test the predictive capacity of the metrics, they included all the metrics into a single model, as it improves the overall model predictive performance [45, 56, 62]. However, researchers typically use only one metric to infer feeding intensity, likely for the sake of simplicity [15, 22, 26]. When using one metric, only a simple linear interpolation of the metric value is needed. In contrast, when combining multiple metrics within the same model, the relative contribution of each metric in explaining feeding intensity variance is required. This statistical information is generally not available to researchers for the species or the ecological context they are studying. Therefore, there is a clear mismatch between how the metrics are tested and how they are used. In addition, previous studies have assumed that the relationship between movement metrics and feeding intensity is the same for all individuals [13, 45, 56, 62]. Nonetheless, variation among individuals in response to environmental conditions (i.e., plasticity) is commonly found in behavioral ecology [63, 64]. Due to these limitations, there is a real need for additional validation of the capacity of movement metrics to infer feeding behavior.

The aim of this study is to quantify and compare the predictive capacity of several continuous track- and dive-based metrics previously proposed to infer feeding intensity (Table 1 provides a descriptive list of the metrics). We conducted this study on female southern elephant seals (SES) from the Kerguelen Archipelago during their post-breeding foraging trips at sea. Female SES undertake foraging trips up to multiple months, which can extend several thousands of kilometers from their haul-out sites [65]. They predominantly forage pelagically in the interfrontal oceanic zone [66–68], where they target oceanic features of higher prey density [69–71] such as (sub)mesoscale eddies and fronts [72–75], the eastward Kerguelen plume [76, 77], and areas with shallower Circumpolar Deep Water [15]. SES exhibit a high segregation among individuals in their core foraging areas characterized by distinct topographic and oceanic features [66, 68, 78] to which they are highly faithful at adult age [79–81]. The diet of SES is predominantly composed of squid and fish [82–85] for which the relative proportion could vary with sex [82], age [86, 87], habitat type [80, 85], season [84], and year [88]. Cherel et al. [89] and Ducatez et al. [90] conducted stable isotope analyses on blood samples of adult female SES from the Kerguelen Islands and concluded that their diets during pre-breeding foraging trips were dominated by a family of small pelagic fish (Myctophidae) regardless of the zones they were foraging in.

**Table 1:** Description of the track and dive-based metrics at the scale of dives

Metric	Description	
Descent rate*	$\frac{{d_{descent} }}{{t_{descent} }}$	Where $d_{descent}$ is the sum of the vertical distance swam during the descent phase and $t_{descent}$ the duration of the descent phase
Ascent rate*	$\frac{{d_{ascent} }}{{t_{ascent} }}$	Where $d_{ascent}$ is the sum of the vertical distance swam during the ascent phase and $t_{ascent}$ the duration of the ascent phase
Bottom duration*		Duration of the bottom phase
Surface duration		Duration of the post-dive surface phase
Efficiency*	$\frac{{t_{bottom} }}{{t_{dive} + t_{surface} }}$	Where $t_{bottom}$ is the duration of the dive bottom phase, $t_{dive}$ the dive duration, and $t_{surface}$ the post-dive surface duration
Sinuosity	$\frac{{d_{bottom} }}{{ld_{bottom} }}$	Where $d_{bottom}$ is the total vertical distance swam by the seal at the bottom phase and $ld_{bottom}$ the sum of the linear vertical distance from the start of the bottom phase to the maximum depth and from the maximum depth to the end of the bottom phase
Hunting time*		Sum of the duration of the dive segments during which the seal is considered as hunting [48, 101]
Horizontal speed	$\frac{\Delta d}{{\Delta t}}$	Where $\Delta d$ is the distance between the current and the previous dive and $\Delta t$ is the time duration between the current and the previous dive
Turning angle		Turning angle between the previous, the current, and the next dive
FPT		The first-passage time method [27]
Move persistence		Correlation in transit speed and turning angle over time [26]

All dive metrics are calculated from the high-resolution dive profiles (i.e., at 1 Hz) and the ones marked with a * are also calculated from the low-resolution dive profiles (i.e., simplified to five segments using the broken stick method similar to data transmitted by CTD-SRDL loggers)

We used the number of prey encounter events (PEE), defined as high bursts of the animal head acceleration, as the reference for feeding attempts from which we infer feeding intensity [10]. The detection of PEE from accelerometry has been a very popular method due to its simplicity, affordability, and minimum invasiveness on animals compared to other available methods [10, 11]. The performance of PEE as a proxy for feeding attempts was initially tested in captivity on hooded seals (*Cystophora cristata*) [10] and Steller sea lions (*Eumetopias jubatus*) [11] by comparing the occurrence of PEE with the true feeding events recorded from video cameras. PEE from accelerometry were also validated on free-ranging animals, for example, on Australian sea lions (*Arctocephalus pusillus doriferus*) [91] and chinstrap (*Pygoscelis antarcticus*) and gentoo penguins (*Pygoscelis papua*) [92]. It was concluded that recorded PEE from accelerometry efficiently detect true PEE but failed to differentiate among prey types and between successful and unsuccessful feeding events [91, 92]. Since then, PEE have been commonly used as a proxy for feeding attempts with numerous marine predators such as SES [44, 70], harbor seals [12], Australian sea lions [8], Antarctic fur seals (*Arctocephalus gazella*) [56], and little penguins (*Eudyptula minor*) [93].

We conducted our analysis on two temporal scales (dive and day), and on two dive data resolutions. We used high-resolution dive profiles sampled at 1 Hz, which we also reduced into five segments (low-resolution dive profiles) using the broken stick method to match dive profiles transmitted by the commonly deployed CTD-SRDL loggers [94]. We also conducted our analysis using two types of models: linear models and regression trees.

## Methods

### Instrument deployment and data collection

**Fig. 1:**
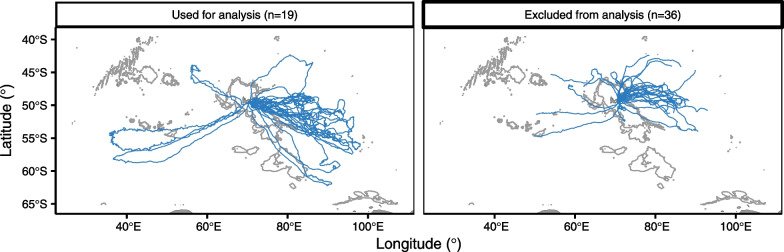
Map of the post-breeding foraging trips of the equipped southern elephant seals (with at least an average of 15 locations per day). The left figure shows the tracks of the seals that were included in the analysis and the right one shows the tracks of the seals that were excluded from the analysis

Between 2010 and 2019, 65 breeding female SES from the Kerguelen Islands (49°20’S, 70°20’E) were equipped by the field crew with loggers before leaving for their post-breeding foraging trips at sea (Fig. 1). Individuals were captured with a head-bag canvas and intravenously sedated with a 1:1 combination of Tiletamine and Zolazepam [Zoletil 100, 95]. All seals were weighed (precision of 0.1 kg) and measured from nose to tail while positioned flat on the ground. After cleaning the fur with acetone, loggers were glued to seals using a quick-setting epoxy [Araldite AW 2101, Ciba, 96]. Individuals were equipped with different logger types and combinations (see Additional file 1 for all details). The data for each seal included either Argos (n = 35) or GPS (n = 30) locations, dive depth (at 0.5 or 1 Hz), and tri-axial acceleration (at 12.5 or 16 Hz). We excluded all data from the first and last day of the trip of each seal.

### Dive data

A dive is defined as any change in depth exceeding 15 m and lasting more than 5 min. We excluded from the analysis any dives with recording errors and outlier behaviors. Although dives with outlier values are not necessarily caused by recording errors, we excluded them as they do not represent the general behavior of the animal and likely have an influential effect on the estimation of the model parameters [97]. A dive is excluded if (1) one or more depth records are missing from the depth profile, (2) two or more depth records occur at the same time, (3) the vertical speed exceed 4.0 m.s^−1^, (4) the dive lasts more than 2800 s, (5) the maximum depth reaches > 1200 m, or (6) the surface time is longer than 300 s. These values were suggested by Cox et al. [98] to identify outlier dives and were confirmed in this study by inspecting the histogram of each variable. We separated dive profiles into three phases: the descent, the bottom, and the ascent phases.

#### High-resolution dive profiles

Using the high-resolution dive profiles (sampled at 1 Hz), we delimited the descent phase from the start of the dive to the first time the vertical speed of the seal reached 0.75 m.s^−1^ [45, 99]. The ascent phase is delimited from the last time the vertical speed of the seal is < 0.75 m.s^−1^ to the end of the dive [45, 99]. The bottom phase is delimited from the end of the descent phase to the start of the ascent phase [45]. From each dive, we extracted the descent and ascent rates, the bottom duration, the post-dive surface duration, the dive efficiency, the bottom phase (vertical) sinuosity, and the hunting time.

The dive efficiency is calculated as the ratio between the bottom phase duration over the sum of the dive and the post-dive surface duration [34]. The bottom phase sinuosity is calculated as the ratio between the total vertical distance traveled by the seal over of the linear vertical distance from the start of the bottom phase to the maximum depth, and from the maximum depth to the end of the bottom phase [45]. Finally, the hunting time is defined as the total time within a dive during which the seal is in hunting mode. Hunting segments are distinguished from transit segments using the method proposed by Heerah et al. [48]. Briefly, the dive profile is segmented using the broken stick method where the number of segments is defined by optimizing the dive zone index [100]. Diving segments are considered as hunting segments when the vertical sinuosity is higher than 1/0.9 and are otherwise defined as transit segments (for all details about this method see [48]). The vertical sinuosity of each dive segment is calculated as the ratio between the total vertical distance traveled by the seal over the linear vertical distance.

#### Low-resolution dive profiles

We reduced the high-resolution dive profiles into five dive segments by identifying four characteristic inflection points via the broken stick algorithm. This reduction in dive profile resolution is intended to match the dive profiles transmitted by CTD-SRDL loggers via the Argos satellite system [94]. From each dive, we extracted (1) the descent and ascent rates, as the ratio between depth and time differences for the first and last dive segments respectively [98], (2) the bottom time as the time between the first and last segments, (3) the dive efficiency, and (4) the hunting time, defined as the total time of all hunting segments. A dive segment is considered a hunting segment when the vertical rate is < 0.4 m.s^−1^, as suggested by Heerah et al. [101]. Dives with inflection points occurring at the same time are removed [98].

### Track data

**Fig. 2:**
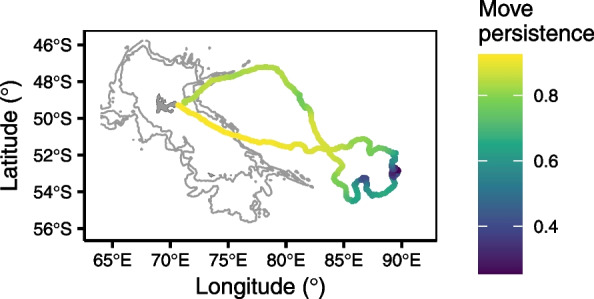
Seal track example color coded according to move persistence from low values (i.e., low movement autocorrelation) in dark blue to high values (i.e., high movement autocorrelation) in yellow

We estimated the location of each dive along the seal track by filtering observed locations with a correlated random walk state-space model that accounts for error in the GPS and Argos system [R package foieGras, 102]. We calculated the following track-based metrics at each dive location: (1) the horizontal speed between the current and the previous dive, (2) the turning angle between the previous, the current, and the next dive, (3) the first-passage time (FPT) as the time required to a seal to exit an area of a given radius [27] for which we set a fixed radius of 25 km to avoid any bias due to between-individual differences in sampling effort, and (4) the move persistence as the autocorrelation in movement (horizontal speed and turning angle) using a state-space model as described in Jonsen et al. [26] with the foieGras R package (Fig. 2). Move persistence models did not always converge when fitted on dive locations; thus, we fitted these models with locations set at a 4 h time step and assigned move persistence values to each dive by linearly interpolating predicted values.

### Prey encounter event

We used prey encounter events (PEE) as reference for feeding attempts which represents feeding activity. PEE are defined as high bursts of head movement that seals perform when attempting to capture a prey [11]. Note that PEE do not distinguish between successful and missed prey captures [91]. We extracted PEE from the raw acceleration data as follow: (1) we separated the dynamic acceleration of the 3 acceleration axes (the animal movement) from the static acceleration (due to gravity) by applying an order 3 high-pass digital Butterworth filter with a normalized cut-off frequency of 0.33 Hz [70]; (2) we reduced and smoothed the resolution of the resulting time series by computing the standard deviation at each second followed by a running standard deviation over a time window of 5 s; (3) we then performed a 2-mean clustering on each axis and defined PEE when the three axes continuously displayed a signal in the cluster with the highest mean value. We considered a PEE distinct from the preceding PEE when separated by at least one second [45].

### Data analysis

To test the capacity of each of the movement metrics (Table 1) to predict nPEE, we fitted a model with each of the metrics. We additionally fitted a model with all the dive-based metrics, one with all the track-based metrics, and one with all the metrics to investigate how the cumulative effect of the metrics impacts the predictive capacity of the model. We conducted our analysis at the scale of dives and days as animals can adjust their behavior differently at short and long temporal scales [61]. At the scale of days, we averaged all the metric values across each day. We used two different types of models to predict nPEE: generalized linear mixed-effect models (GLMM) and boosted regression tree models (BRT). GLMM are widely used in ecology to model behavior (e.g., [45, 62]) and allow to decompose the total phenotypic variance into different hierarchical levels, e.g., among and within individuals [103]. BRT are popular for their predictive robustness as they are not restricted by any distributional or independency assumptions of the data and implicitly account for nonlinearity and interactions in the relationships between predictors and the response variable [104].

We fitted the GLMM using the R package glmmTMB [104] with nPEE as the response variable and the metrics as fixed effects. We used a Poisson distribution with a log link function. We allowed the intercepts and the slopes between nPEE and the metrics to vary among individuals (i.e., random effects). All metrics were normalized (i.e., mean-centered and unit variance). All details about constructing and checking the GLMM are presented in the Additional file 2.

Based on the framework described in Rights & Sterba [105], we partitioned the proportion of the total variance in nPEE explained by the GLMM (i.e., the coefficient of determination; ${R}^{2}$) into the proportion explained by the predictors via the fixed slope variance (${R}_{F}^{2}$, ${R}_{t}^{2\left({f}_{1}\right)}$ in [105]), the proportion explained by the individual-specific means via the random intercept variance (${R}_{I}^{2}$, ${R}_{t}^{2\left(m\right)}$ in [105]), and the proportion explained by the predictors via the random slope variance/covariance (${R}_{S}^{2}$, ${R}_{t}^{2\left(v\right)}$ in [105]). We computed the total variance for a Poisson GLMM following Nakagawa et al. [106].

We fitted the BRT using the R package xgboost [107] with nPEE as the target variable and the metrics as the predictors. We used the tweedie distribution as the objective of the model as it is suitable for modeling dispersion and accounting for zero-inflation [108]. To improve the model predictive performance, we tuned several hyperparameters of each of the models (see all details in the Additional file 3).

The predictive capacity of each of the models was evaluated from the accuracy and the correlation between the predicted and the observed nPEE for each individual using a leave-one-individual-out cross-validation procedure (e.g., [45, 62]). We iteratively excluded each individual seal from the dataset, refitted the model with the remaining individuals, and calculated the accuracy and the correlation between the observed and the predicted values of the excluded individual. We quantified the accuracy of the models using the root-mean-square error (RMSE) such as:

$${\text{RMSE}} = \sqrt {\frac{{\mathop \sum \nolimits_{t = 1}^{n} \left( {\hat{y}_{t} - y_{t} } \right)^{2} }}{n}}$$

where $\widehat{y}$ is the predicted value, $y$ is the observed value, and $n$ is the number of observations. To compare the accuracy of the models at the scale of dives with the models at the scale of days, we also computed two normalized versions of RMSE: the mean-based normalized RMSE ($mRMSE = RMSE/\overline{y}$) and the standard-deviation-based normalized RMSE ($sdRMSE = RMSE/\sigma_{y}$). We then compared the models such as the models with the highest predictive capacity have the lowest RMSE, mRMSE, sdRMSE, and the highest positive correlation coefficients. When the model is fitted with GLMM, the model performance is also represented by a large amount of the variance in nPEE explained by the fixed effects while minimizing the among-individual variance. We conducted our analysis on R 4.1.3 [109].

## Results

As most of the devices stopped recording before the end of the foraging trips, seals varied substantially in the duration of the recorded data, ranging from 11 to 84 days. To minimize any bias in representing the behavior of the seals, we retained in our analysis only the seals with at least 30 days of recording data. We ended up using 21 out 65 of the female SES, for which we had on average 71 ± 8 days of data (range: 53–83 days). The seals weighed 289 ± 63 kg (mean ± sd; range: 200–413 kg) and measured 2.39 ± 0.21 m (range: 2.06–2.84 m, Additional file 1). After filtering the data, we analyzed 100,931 dives, from which 88 ± 4% had at least one PEE. Seals performed 9 ± 8 (max: 45) PEE per dive and 653 ± 315 (max: 1,755) PEE per day. Some seals had a low number of locations per day; therefore, we excluded these individuals (n = 2) from all models that involved track-based metrics by using a threshold of an average of 15 locations per day. This threshold was defined visually from the histogram of the mean number of locations of all the seals (Additional file 4).

### Model predictive performance

**Fig. 3:**
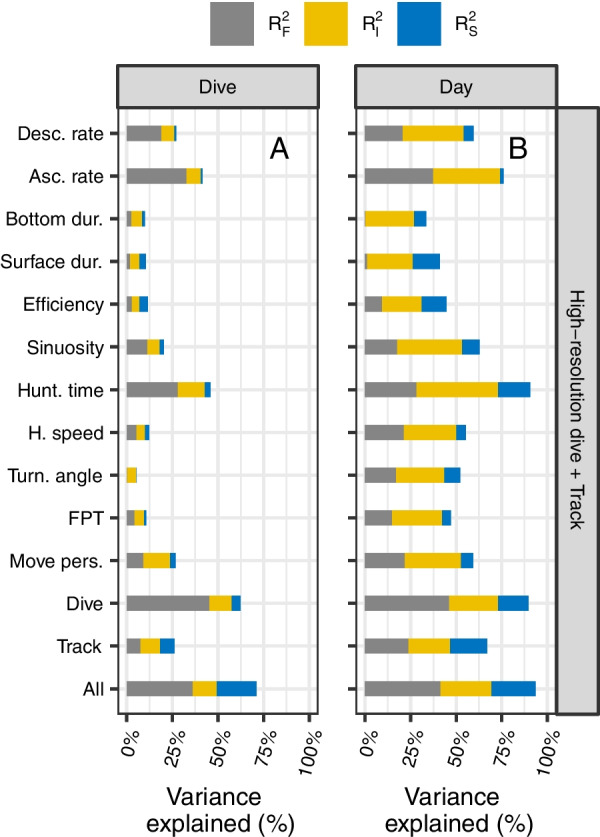
Partitioning of the variance in the number of prey encounter events (nPEE) explained by each of the GLMM (on the y-axis) at the scale of dives (**A**) and days (**B**). The proportion of the variance explained by the predictors via the fixed slope variance (${{R}}_{{{F}}}^{2}$; grey bar), by the individual-specific means via the random intercept variance (${{R}}_{{{I}}}^{2}$; yellow bar), and by the predictors via the random slope variance/covariance (${{R}}_{{{S}}}^{2}$; blue bar). The dive model includes all the dive-based metrics, the track model includes all the track-based metrics, and the all model includes all the metrics. Dive metrics are calculated from the high-resolution dive profiles (i.e., at 1 Hz)

**Fig. 4:**
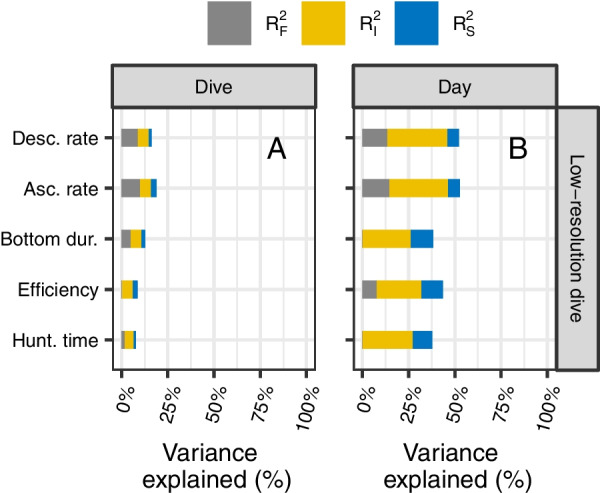
Partitioning of the variance in the number of prey encounter events (nPEE) explained by each of the GLMM (on the y-axis) at the scale of dives (**A**) and days (**B**). The proportion of the variance explained by the predictors via the fixed slope variance (${{R}}_{{{F}}}^{2}$; grey bar), by the individual-specific means via the random intercept variance (${{R}}_{{{I}}}^{2}$; yellow bar), and by the predictors via the random slope variance/covariance (${{R}}_{{{S}}}^{2}$; blue bar). Dive metrics are calculated from the low-resolution dive profiles, i.e., simplified to five segments using the broken stick method similar to data transmitted by CTD-SRDL loggers

Regardless of the temporal scale, the dive profile resolution, and the type of the model used, the model including the ascent rate best predicted nPEE ($R_{{F\left( {dive} \right)}}^{2}$ = 33% at the scale of dives and $R_{{F\left( {day} \right)}}^{2}$ = 37% at the scale of days), closely followed by the model including the hunting time ($R_{{F\left( {dive} \right)}}^{2}$ = 28% and $R_{{F\left( {day} \right)}}^{2}$ = 28%), and the descent rate ($R_{{F\left( {dive} \right)}}^{2}$ = 19% and $R_{{F\left( {day} \right)}}^{2}$ = 21%, Fig. 3, Fig. 4, Table 2, and Table 3).

**Table 2:** Performance output of the track- and dive-based metrics using GLMM in explaining and predicting nPEE at the scale of dives and days

Model	R2F (%)	R2I (%)	R2S (%)	RMSE	mRMSE	sdRMSE	Corr	R2F (%)	R2I (%)	R2S (%)	RMSE	mRMSE	sdRMSE	Corr
	Dive							Day						
Descent rate	19	7	1	7.4 ± 2.2	0.78 ± 0.10	1.05 ± 0.12	0.31 ± 0.12	21	34	5	308.6 ± 134.6	0.49 ± 0.19	1.33 ± 0.48	0.41 ± 0.29
Ascent rate	33	8	1	7.2 ± 2.1	0.77 ± 0.14	1.02 ± 0.14	0.46 ± 0.08	37	37	2	313.9 ± 165.3	0.50 ± 0.24	1.36 ± 0.65	0.61 ± 0.17
Bottom duration	3	6	1	7.6 ± 2.3	0.81 ± 0.11	1.08 ± 0.08	0.08 ± 0.12	0	27	7	299.9 ± 136.1	0.46 ± 0.16	1.24 ± 0.24	0.03 ± 0.32
Surface duration	2	5	3	7.6 ± 2.2	0.81 ± 0.09	1.08 ± 0.07	0.11 ± 0.14	1	25	15	292.6 ± 134.6	0.45 ± 0.13	1.20 ± 0.18	0.13 ± 0.31
Efficiency	3	4	4	7.6 ± 2.1	0.81 ± 0.08	1.08 ± 0.06	0.17 ± 0.17	9	22	13	277.2 ± 112.2	0.43 ± 0.13	1.17 ± 0.21	0.37 ± 0.33
Sinuosity	11	7	2	7.5 ± 2.2	0.80 ± 0.11	1.07 ± 0.09	0.21 ± 0.17	18	36	10	325.8 ± 134.5	0.52 ± 0.19	1.43 ± 0.52	0.37 ± 0.35
Hunting time	28	15	3	10.1 ± 4.3	1.14 ± 0.68	1.52 ± 0.88	0.37 ± 0.11	28	45	18	861.6 ± 668.2	1.52 ± 1.53	4.45 ± 4.76	0.45 ± 0.32
Horizontal speed	5	5	2	7.8 ± 2.2	0.81 ± 0.10	1.07 ± 0.07	0.18 ± 0.11	21	29	5	300.2 ± 122.7	0.48 ± 0.22	1.26 ± 0.45	0.50 ± 0.24
Turning angle	0	5	0	7.9 ± 2.2	0.82 ± 0.10	1.08 ± 0.07	0.02 ± 0.04	17	27	9	389.1 ± 224.1	0.66 ± 0.61	1.69 ± 1.16	0.30 ± 0.17
FPT	4	5	1	7.7 ± 2.1	0.81 ± 0.10	1.07 ± 0.06	0.15 ± 0.09	15	28	5	322.4 ± 111.3	0.52 ± 0.21	1.36 ± 0.43	0.38 ± 0.19
Move persistence	9	15	3	8.0 ± 2.0	0.84 ± 0.10	1.10 ± 0.07	0.17 ± 0.11	22	31	7	313.8 ± 90.9	0.49 ± 0.15	1.32 ± 0.33	0.42 ± 0.24
Dive	45	13	5	7.1 ± 2.0	0.77 ± 0.13	1.03 ± 0.18	0.59 ± 0.07	46	27	17	282.0 ± 147.8	0.45 ± 0.24	1.25 ± 0.74	0.81 ± 0.11
Track	7	11	8	7.8 ± 2.1	0.82 ± 0.09	1.08 ± 0.07	0.20 ± 0.10	24	23	20	291.5 ± 109.7	0.46 ± 0.16	1.20 ± 0.32	0.53 ± 0.22
All	36	13	22	7.8 ± 2.1	0.82 ± 0.13	1.09 ± 0.21	0.58 ± 0.07	41	28	24	324.2 ± 158.6	0.50 ± 0.23	1.38 ± 0.74	0.81 ± 0.12
Descent rate	9	6	1	7.5 ± 2.4	0.79 ± 0.11	1.05 ± 0.10	0.22 ± 0.12	13	33	6	311.4 ± 137.1	0.49 ± 0.18	1.32 ± 0.35	0.31 ± 0.29
Ascent rate	10	6	3	7.5 ± 2.5	0.79 ± 0.12	1.05 ± 0.12	0.26 ± 0.14	14	32	6	316.0 ± 162.5	0.48 ± 0.17	1.30 ± 0.35	0.28 ± 0.27
Bottom duration	5	6	2	7.7 ± 2.3	0.81 ± 0.11	1.08 ± 0.08	0.10 ± 0.13	0	26	12	301.6 ± 138.8	0.46 ± 0.15	1.24 ± 0.22	−0.05 ± 0.35
Efficiency	0	6	3	7.7 ± 2.2	0.82 ± 0.09	1.09 ± 0.07	0.12 ± 0.15	8	24	12	279.9 ± 122.0	0.43 ± 0.13	1.17 ± 0.22	0.34 ± 0.35
Hunting time	2	5	1	7.6 ± 2.1	0.81 ± 0.09	1.07 ± 0.07	0.14 ± 0.11	0	27	10	299.9 ± 132.7	0.47 ± 0.17	1.25 ± 0.25	−0.00 ± 0.38

The variance in nPEE explained by the GLMM (each row) is partitioned into the proportion of variance explained by the predictors via the fixed slope variance ($R_{F}^{2}$), by the individual-specific means via the random intercept variance ($R_{I}^{2}$), and by the predictors via the random slopes variance/covariance ($R_{S}^{2}$). The root-mean-square error (RMSE; mean $\pm$ sd), the mean-based normalized RMSE (mRMSE), the standard-deviation-based normalized RMSE (sdRMSE), and the correlation coefficient (Corr.) are computed between the observed values of nPEE for each seal and the predicted values by the model fitted without the focal individual (i.e., leave-one-individual-out cross-validation). The dive model includes all the dive-based metrics, the track model includes all the track-based metrics, and the all model includes all the metrics. Dive metrics are calculated from the high-resolution dive profiles (i.e., at 1 Hz; upper section) or from the low-resolution dive profiles (lower section), i.e., simplified to five segments using the broken stick method similar to data transmitted by CTD-SRDL loggers.

**Table 3:** Performance output of the track- and dive-based metrics using BRT in explaining and predicting nPEE at the scale of dives and days

Model	RMSE	mRMSE	sdRMSE	Corr	RMSE	mRMSE	sdRMSE	Corr
	Dive				Day			
Descent rate	6.7 ± 1.8	0.72 ± 0.10	0.96 ± 0.05	0.35 ± 0.08	242.6 ± 125.4	0.36 ± 0.11	0.98 ± 0.16	0.38 ± 0.29
Ascent rate	6.4 ± 1.8	0.69 ± 0.11	0.91 ± 0.06	0.49 ± 0.07	245.0 ± 131.6	0.37 ± 0.11	0.98 ± 0.17	0.49 ± 0.19
Bottom duration	7.0 ± 1.8	0.75 ± 0.11	0.99 ± 0.03	0.22 ± 0.08	260.6 ± 127.7	0.39 ± 0.11	1.05 ± 0.13	0.16 ± 0.28
Surface duration	7.1 ± 1.7	0.76 ± 0.09	1.01 ± 0.02	0.10 ± 0.14	259.2 ± 115.7	0.39 ± 0.09	1.07 ± 0.08	0.10 ± 0.23
Efficiency	6.9 ± 1.6	0.75 ± 0.10	0.99 ± 0.04	0.22 ± 0.15	239.0 ± 100.6	0.37 ± 0.09	1.00 ± 0.15	0.35 ± 0.31
Sinuosity	6.8 ± 1.9	0.73 ± 0.10	0.97 ± 0.05	0.31 ± 0.12	256.6 ± 127.8	0.38 ± 0.10	1.04 ± 0.13	0.29 ± 0.28
Hunting time	6.6 ± 1.9	0.71 ± 0.10	0.94 ± 0.06	0.50 ± 0.07	258.7 ± 120.7	0.39 ± 0.10	1.06 ± 0.14	0.02 ± 0.45
Horizontal speed	7.2 ± 1.8	0.76 ± 0.10	1.00 ± 0.03	0.18 ± 0.10	245.7 ± 123.6	0.37 ± 0.11	0.96 ± 0.15	0.51 ± 0.23
Turning angle	7.3 ± 1.8	0.77 ± 0.10	1.01 ± 0.03	0.03 ± 0.04	262.4 ± 115.1	0.39 ± 0.09	1.04 ± 0.11	0.28 ± 0.19
FPT	7.1 ± 1.7	0.75 ± 0.10	0.99 ± 0.03	0.22 ± 0.09	231.8 ± 110.8	0.35 ± 0.10	0.91 ± 0.14	0.56 ± 0.22
Move persistence	7.3 ± 1.7	0.77 ± 0.11	1.01 ± 0.02	0.14 ± 0.10	250.5 ± 100.2	0.38 ± 0.09	1.00 ± 0.09	0.43 ± 0.26
Dive	5.6 ± 1.2	0.61 ± 0.09	0.81 ± 0.07	0.62 ± 0.10	174.1 ± 83.1	0.27 ± 0.09	0.73 ± 0.17	0.74 ± 0.13
Track	7.2 ± 1.7	0.76 ± 0.10	1.00 ± 0.03	0.22 ± 0.07	234.7 ± 90.9	0.36 ± 0.08	0.95 ± 0.13	0.49 ± 0.25
All	5.8 ± 1.3	0.61 ± 0.09	0.81 ± 0.06	0.62 ± 0.09	183.6 ± 76.6	0.28 ± 0.08	0.74 ± 0.14	0.74 ± 0.15
Descent rate	7.0 ± 1.9	0.75 ± 0.10	1.00 ± 0.04	0.22 ± 0.10	255.8 ± 133.3	0.38 ± 0.11	1.03 ± 0.16	0.24 ± 0.26
Ascent rate	7.0 ± 1.9	0.74 ± 0.10	0.99 ± 0.05	0.26 ± 0.11	265.8 ± 133.2	0.40 ± 0.10	1.07 ± 0.14	0.03 ± 0.25
Bottom duration	7.0 ± 1.9	0.75 ± 0.11	1.00 ± 0.03	0.21 ± 0.09	262.4 ± 127.2	0.39 ± 0.11	1.06 ± 0.13	0.06 ± 0.33
Efficiency	7.0 ± 1.7	0.76 ± 0.10	1.01 ± 0.03	0.14 ± 0.14	242.1 ± 111.9	0.37 ± 0.10	1.00 ± 0.16	0.30 ± 0.35
Hunting time	6.9 ± 1.7	0.74 ± 0.10	0.98 ± 0.03	0.26 ± 0.10	261.0 ± 127.2	0.39 ± 0.10	1.06 ± 0.12	0.14 ± 0.22

The root-mean-square error (RMSE; mean ± sd), the mean-based normalized RMSE (mRMSE), the standard-deviation-based normalized RMSE (sdRMSE), and the correlation coefficient (Corr.) are computed between the observed values of nPEE for each seal and the predicted values by the model fitted without the focal individual (i.e., leave-one-individual-out cross-validation). The dive model includes all the dive-based metrics, the track model includes all the track-based metrics, and the all model includes all the metrics. Dive metrics are calculated from the high-resolution dive profiles (i.e., at 1 Hz; upper section) or from the low-resolution dive profiles (lower section), i.e., simplified to five segments using the broken stick method similar to data transmitted by CTD-SRDL loggers

#### GLMM vs BRT

**Fig. 5:**
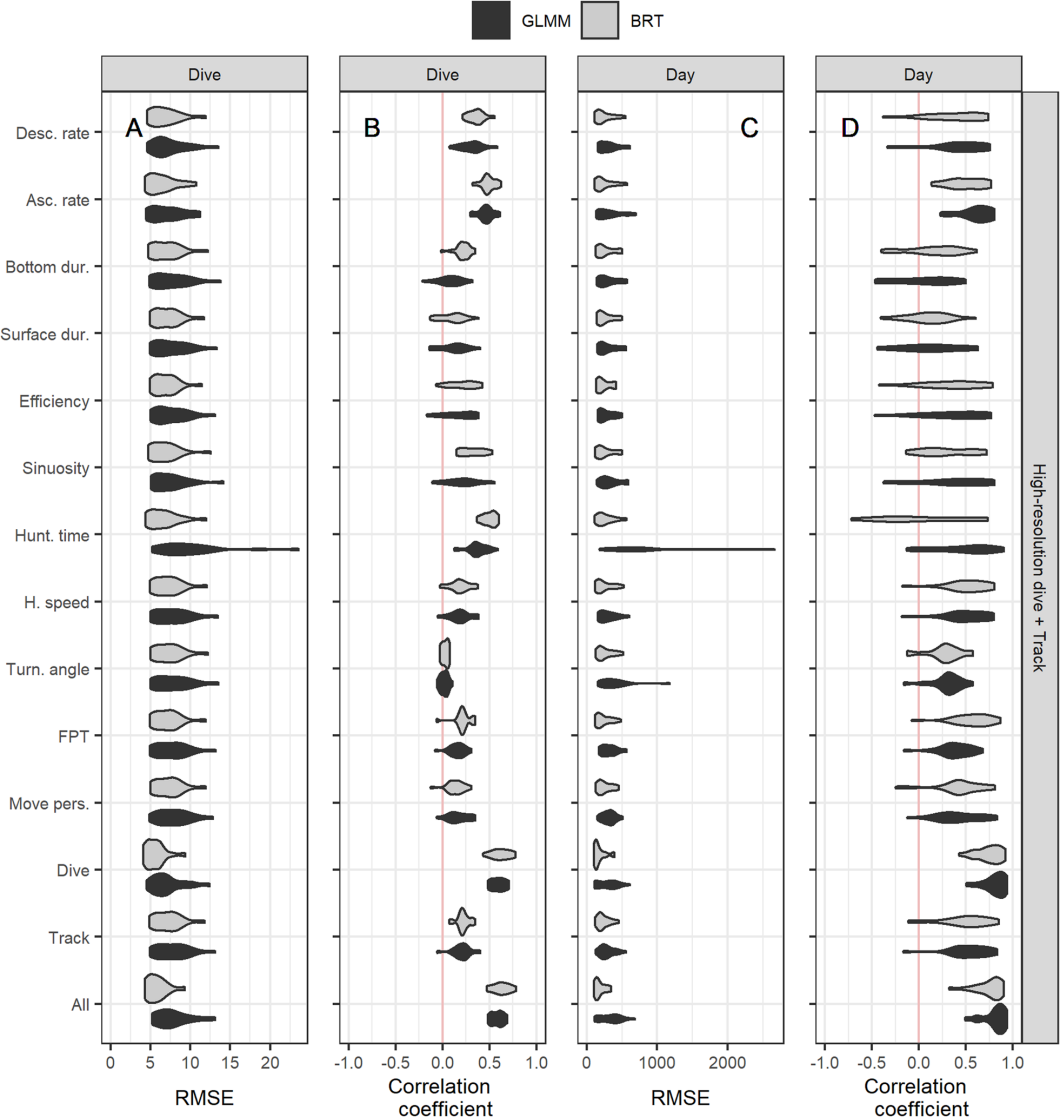
Performance of each of the models (on the y-axis) in predicting the number of prey encounter events (nPEE) at the scale of dives (**A** and **B**) and days (**C** and **D**). Each of the models is fitted with a generalized linear mixed-effect model (GLMM; in black) and with a boosted regression tree (BRT; in grey). The root-mean-square error (RMSE) and the correlation coefficient are computed between the observed values of nPEE for each seal and the predicted values by the model fitted without the focal individual (i.e., leave-one-individual-out cross-validation). The dive model includes all the dive-based metrics, the track model includes all the track-based metrics, and the all model includes all the metrics. Dive metrics are calculated from the high-resolution dive profiles (i.e., at 1 Hz)

**Fig. 6:**
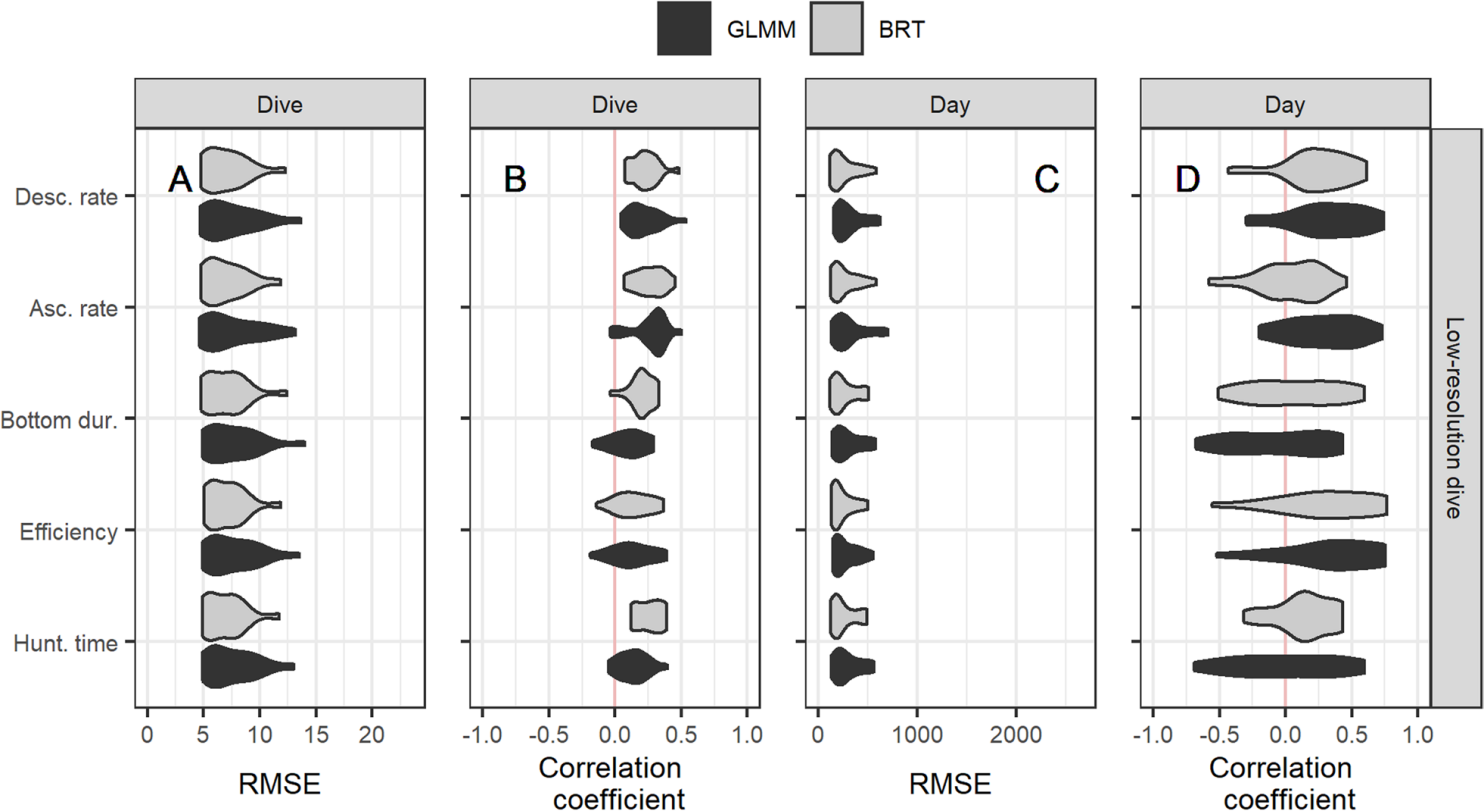
Performance of each of the models (on the y-axis) in predicting the number of prey encounter events (nPEE) at the scale of dives (**A** and **B**) and days (**C** and **D**). Each of the models is fitted with a generalized linear mixed-effect model (GLMM; in black) and with a boosted regression tree (BRT; in grey). The root-mean-square error (RMSE) and the correlation coefficient are computed between the observed values of nPEE for each seal and the predicted values by the model fitted without the focal individual (i.e., leave-one-individual-out cross-validation). Dive metrics are calculated from the low-resolution dive profiles, i.e., simplified to five segments using the broken stick method similar to data transmitted by CTD-SRDL loggers

All BRT models were more accurate and generally had higher correlations than GLMM. However, at the scale of days, it was not clear whether BRT or GLMM performed better based on the mean correlation values (Fig. 5, Fig. 6, Table 2, and Table 3). The model including the hunting time had extreme high values of RMSE for some individuals when fitted with GLMM and not when fitted with BRT (Fig. 5). Output estimates of all GLMM are presented in the Additional file 5.

#### Single vs multiple metrics

Models that included all the dive-based metrics ($R_{{F\left( {dive} \right)}}^{2}$ = 45% and $R_{{F\left( {day} \right)}}^{2}$ = 46%) performed better than the model that included all the track-based metrics ($R_{{F\left( {dive} \right)}}^{2}$ = 7% and $R_{F}^{{2\left( {day} \right)}}$ = 24%) and any model that included a single metric. The model that included all the metrics performed similarly to the model that included all the dive-based metrics (Fig. 3, Fig. 5, Table 2, and Table 3).

#### Low vs high-resolution dive profiles

All dive-based metrics computed from the low-resolution dive profiles explained less or similar mean effect variance ($R_{F}^{2}$) than the models including the metrics computed from the high-resolution dive profiles (Fig. 3, Fig. 4, & Table 2). The models that included the ascent rate, the descent rate, or the hunting time showed the most pronounced decrease in the predictive capacity when computed with the low-resolution dive profiles compared to the high-resolution dive profiles.

#### Scale of dives vs days

GLMM with a single metric explained between 0 and 33% of the variance in nPEE at the scale of dives, and between 0 and 37% of the variance in nPEE at the scale of days (Table 2). At the scale of days, all variance components tended to increase (Fig. 3 and Table 2). This increase in variance components was particularly marked in the among-individual variances ($R_{I}^{2}$ and $R_{S}^{2}$). Higher variation among individual at the scale of days resulted in higher variability among individuals in the correlation values (Fig. 5, Table 2, and Table 3). The models that included the dive bottom duration, the post-dive surface duration, and the hunting time were the only models that did not show a substantial increase in $R_{F}^{2}$ at the scale of days compared to the scale of dives (Fig. 3 and Table 2). At the scale of days, the GLMM including hunting time computed from high-resolution dive profiles had the largest value of $R_{I}^{2}$ = 45% compared to other models (Fig. 3). Models including one of the dive-based metrics increased more in $R_{S}^{2}$ from the scale of dives to the scale of days compared to the models including one of the track-based metrics. For all models, mRMSE values at the scale of dives were larger than mRMSE at the scale of days, whereas sdRMSE values at the scale of days were larger than sdRMSE at the scale of dives for GLMM and similar for BRT.

## Discussion

We tested and compared a series of track- and dive-based movement metrics in how well they predict feeding intensity in SES, which was inferred from nPEE measured with accelerometry. We found that none of the metrics predicted nPEE with a high accuracy and correlation (i.e., > 0.5) with the observed nPEE in all individual seals. The performance of the metrics varied largely among individuals, especially at the scale of days, where some individuals had high positive correlations and others had low or negative correlations between the observed and predicted nPEE. Most of the metrics explained a small proportion of the population variance, in addition to a substantial among-individual variance. Although our results may not be representative of other situations involving different species or ecological contexts, we advocate that the complexity of factors driving animal movement is likely ubiquitous [7, 70, 110]. We therefore stress that the utilization of simple movement metrics to infer feeding activity, in particular with diving predators, should be carefully tested a priori (e.g., in pilot studies with high resolution data) during which the most influential factors should be identified and accounted for in subsequent studies, or otherwise highly biased inferences should be expected.

### Dive-based metrics

Among all metrics tested, both transit rate metrics (i.e., ascent rate and descent rate) were the best metrics in predicting the variance of nPEE, regardless of the temporal scale, the resolution of the dive profiles, and the model type used. This important contribution of transit rates in the seal behavioral response to prey encounter was also found in other studies on SES [13, 45, 58, 62] and other diving species [8, 40, 51, 54, 56]. This result is consistent with optimal diving theoretical models predicting that the transit time has a substantial effect on diving success [111]. However, the dive efficiency metric predicted nPEE poorly, at least at the scale of dives, which suggests that the main motivational objective of the seals at short temporal scales may not be to maximize time at the bottom phase over the total dive cycle as predicted by the optimal diving theory [34, 36]. Then, why do seals alter their vertical transit behavior in response to prey density if it is not to maximize time at the foraging phase, i.e., the dive bottom phase?

One possible explanation is that seals increase transit rates to avoid losing contact with a prey patch previously found [112]. In several diving species, individuals increase vertical transit rates by steeper descent and ascent angles, rather than higher swimming speeds, when encountering higher prey density, allowing them to return more rapidly to the same foraging spot with minimal energy expenditure [13, 40, 42]. Sato et al. [41] hypothesized that macaroni penguins (*Eudyptes chrysolophus*) adopt steep body angles during descent and ascent phases and increase time at the bottom of the dive when encountering prey patches, and otherwise adopt shallow body angles and short bottom times to move horizontally more efficiently and increase the probability of locating a good prey patch. Nonetheless, the behavior of divers during the transit phase depends on different factors, which makes it hard to tease apart all sources of variance. For example, swimming speed during transit phases in grey seals increased with distance to prey patches [113] but decreased in northern elephant seals with depth [114]. Moreover, buoyancy affects swimming speed, stroke rate, and gliding behavior during transit phases both in northern [115] and southern elephant seals [116].

Although the dive bottom duration has been used as a proxy for feeding activity [117–119], we found in SES that the bottom duration is a poor predictor for nPEE. This could be explained by the multiple factors affecting how divers adjust their dive bottom duration. For example, divers may alter their dive bottom duration in response to the interaction between body buoyancy and mass, prey distribution in space and time, and the depth at which prey patches are found [53, 57, 58, 61, 99]. As SES dive continuously during their time at sea, they also perform non-feeding dives, such as rest and exploration dives [120], which may add noise in the variation of the dive bottom duration, reducing its power to predict nPEE during feeding dives. However, the dive bottom duration seems more reliable in distinguishing feeding versus non-feeding dives rather than the density of prey encountered [44, 57, 99].

In contrast, the hunting time computed from high-resolution dive profiles, which captures the variability of the vertical movement of the seal at the bottom phase, performed better than the bottom duration, the dive efficiency, or the overall bottom dive sinuosity. This result is consistent with several previous findings [13, 49, 50]. For example, SES exhibit horizontally and vertically sinuous movements at the bottom phase when encountering prey items [13]. However, the performance of the hunting time metric in explaining the variance in nPEE remains poor ($R_{{F\left( {dive} \right)}}^{2}$ = 28% and $R_{{F\left( {day} \right)}}^{2}$ = 28%). SES adopt different hunting modes involving either active-swimming approaches or passive-gliding approaches from above the prey [121]. Jouma’a et al. [121] found, in six Kerguelen female SES, that passive-gliding approaches occurred ca. 30% of the prey capture attempts, which may weaken the relationship between the hunting time and nPEE.

We found that the ascent rate is a better predictor for nPEE than the descent rate. This can be explained by the effect of the seal buoyancy on its swimming behavior. After the breeding period on land, female SES are in poor condition (i.e., low in fat composition), and hence, they are negatively buoyant when leaving the Kerguelen islands [116, 122]. When negatively buoyant, seals tend to glide down to the bottom of the dive while swimming actively to return to the surface [13, 114–116, 122]. Seals may adjust the duration of the ascent phase more to improve foraging output while minimizing the cost of transport during the descent phase [115], which lead to more variability in descent rate compared to ascent rate [116]. For example, the buoyancy of elephant seals affects swimming speed variability during the descent phase and not during the ascent phase [116, 123]. However, when the seal buoyancy becomes positive after some time foraging at sea, seals tend to glide up to the surface during the ascent phase [124]. In this case, we expect that descent rate will overcome the ascent rate in predicting nPEE. Additionally, divers are assumed to adjust the descent phase in response to prey encountered in previous dives as an anticipatory mechanism [41, 44, 93]. However, divers may be constantly in a searching mode while descending to reach prey patches that are heterogeneously distributed in depth [34, 41], which may contribute to the poorer relationship between the descent rate and nPEE compared to the ascent rate.

### High vs low-resolution dive profiles

All metrics that were calculated from the low-resolution dive profiles performed less well at predicting nPEE than their equivalent metrics calculated from the high-resolution dive profiles. This reduction in performance was especially pronounced in the metrics that performed the best when computed from high-resolution dive profiles such as the descent rate, the ascent rate, and the hunting time. Dive profiles of diving predators like SES can be complex, and defining the descent and the ascent (transit) phases is not always straightforward [125, 126]. Transit phases in SES generally last several minutes and therefore seals are likely to encounter prey on which they opportunistically feed [99]. These interruptions in transit phases can add considerable noise into metrics like the descent and ascent rates depending on how these phases are delimited. In this study, we considered the descent and the ascent phases in high-resolution dive profiles as the first and the last dive segments where the vertical speed of the seal is uninterrupted, i.e., above a certain rate threshold. Although we believe this method is appropriate to estimate transit rates, it might result in underestimating the duration of the transit phases when these phases are composed by subphases. This may result in impacting the values of metrics such as the bottom duration, the bottom phase sinuosity, or the dive efficiency. The broken-stick algorithm, used for the segmentation of the low-resolution dive profiles, breaks-down the dive profiles into five segments based on the general shape of the dive [94]. With this method, there is no guaranty that the first and the last segments of the broken-stick algorithm match with the true descent and ascent phases, which is likely to mismatch with complex dive profiles [see Fig. 1 in Heerah et al., 127].

The hunting time metric was developed initially to distinguish hunting segments from transit segments within a dive, as these hunting segments include most of the PEE [48, 101]. However, the relationship between the duration, or other characteristics (e.g., vertical rate), of these hunting segments and feeding activity was never explicitly tested. Despite this lack of validation, several studies used hunting time as a proxy for prey density, foraging success, or foraging effort [15, 74, 128, 129]. Moreover, the hunting time metric was tested only on few individuals [101, 127], while our results show that the performance of all metrics varies substantially among individuals. Thus, the initial validation of the hunting time to infer feeding behavior is likely biased towards the behavior of some individuals in the population.

### Track-based metrics

Although the ARS behavior matches with feeding activity in different studies on diving species [19, 130–132], we found that all track-based metrics performed poorly in predicting nPEE in SES, and this result was more pronounced at the scale of dives than the scale of days. This is consistent with the study conducted by Vacquié-Garcia et al. [45], where track-based metrics did not explain much of the variance in nPEE after accounting for dive-based metrics. The omnipresence of the ARS hypothesis in marine foraging predators remains questionable as many studies also failed to validate it [55, 58, 133]. For example, southern bluefin tuna (*Thunnus maccoyiii*) and Adélie penguins do not fit the traditional ARS framework; instead, they intensify feeding activity during linear and fast-transit phases compared to the tortuous and slow-transit phases that were hypothesized as resting periods [134–136].

The poor performance of the SES horizontal movement in predicting nPEE may arise from several factors. Della Penna et al. [137] described SES movements as “quasi-planktonic”, i.e., drifting with ocean currents, which may allow seals to dedicate most of their energy in diving and capturing prey instead of moving at the horizontal dimension. Foraging predators may adopt an ARS tactic only at a specific spatial or temporal scale [19], and the scale level may vary among individuals due to the local prey distribution [118] or to individual specialization in foraging tactics [138]. Also, the track data is generally in lower resolution than the dive data. All these potential explanations are supported, but not teased apart, by the fact that the models including track-based metrics explained a higher $R_{F}^{2}$ at the scale of days. At a lower temporal scale, the track data may not be able to capture the horizontal movement of the animal due to the added noise from ocean currents and the data resolution itself, or seals may adopt an ARS behavior only at higher spatiotemporal scales.

**Fig. 7:**
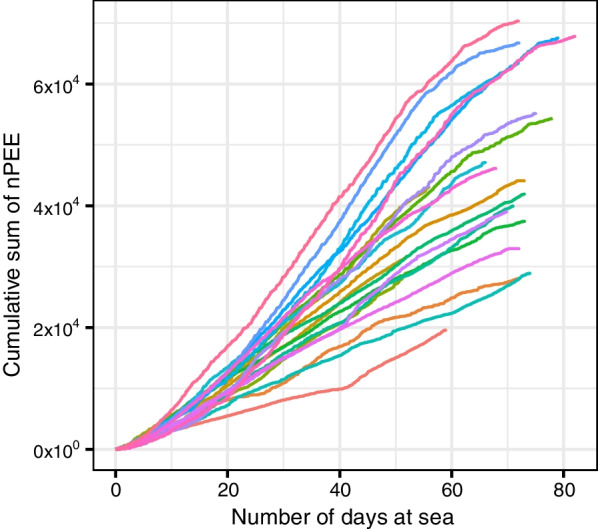
The cumulative sum of nPEE over the foraging trip at sea of the 21 seals used for the analysis

During the post-breeding foraging trips, female SES are in a poor body condition, and thus require a rapid energy intake to avoid mortality. As SES forage in an unpredictable and heterogenous three-dimensional environment, there may be a trade-off in the horizontal movement patterns adopted depending on the motivational objective of the seals. ARS behavior may not be optimal in this context, as the seals must quickly supply elevated energy requirements as opposed to maximizing their long-term energy intake [135, 139]. The plot of the cumulative sum of nPEE over time shows that seals feed continuously (Fig. 7). Hence, feeding continuously and opportunistically may be more efficient to increase survival probability until seals improve their body condition to a certain level. This foraging movement behavior is also more efficient than the ARS behavior when prey are widely dispersed in the environment [140, 141]. This effect may also emerge due to a temporal sampling design biased towards the first part of the foraging trip at sea, where all seals were sampled at the beginning of their trip at sea, right after the breeding season, but varied in the total proportion of the trip that is sampled.

### Foraging temporal scale

All movement metrics performed better at the scale of days than at the scale of dives, which is consistent with previous findings [45, 56]. However, the degree of improvement of the bottom and surface durations was very minimal compared to other metrics. This general improvement in prediction performance, particularly the mean dive efficiency, suggests that seals adjust their diving behavior to optimize foraging success at a larger temporal scale than the dive *per se*. Accounting for the temporal scales at which a forager alters its behavior to optimize benefits and costs is necessary for fully understanding foraging behavior [142, 143]. Rate maximization may operate simultaneously on several (or all) temporal scales where distinct currencies are targeted at each scale [117, 144]. Therefore, the relationship between movement metrics and prey density can be scale-dependent [59, 61]. For example, Adélie penguins increase dive bottom duration in response to krill capture rate at the scale of dives, and they decrease it at the scale of bouts [61]. Although scale-specific behavioral adjustments make up for different motivational objectives, they come with physiological and behavioral constraints resulting in foraging scale trade-offs. For example, bison (*Bison bison*) prefer to optimize their short-term energy gains at the expense of long-term gains by foraging on *Carex atherodes* instead of *Agropyron* spp., presumably due to the risk of predation and anthropogenic disturbance [145].

### Inter-individual variability

We found that a substantial proportion of the variance in nPEE is explained by individual differences (between 5 and 18% at the scale of dives and between 33 and 63% at the scale of days), which is commonly found in SES behavior [138, 146] as well as in other diving predators [147–149]. Among-individual variance includes the variance due to differences in the mean nPEE, differences in how seals adjust their behavior in response to nPEE, and the covariance between both [105]. All variance components explained by among-individual differences were larger at the scale of days than at the scale of dives. This suggests again that seals adjust their foraging behavior at larger temporal scales. The among-individual variance could be explained by many extrinsic and intrinsic factors.

When leaving the Kerguelen Islands, SES seals spread in all directions in the Indian section of the Southern Ocean and consequently forage in areas with varying conditions [Fig. 1, 67, 68, 78]. This likely results in targeting different types of prey with varying characteristics such as size, energetical and nutritional content, accessibility, and digestibility [80, 85]. A recent study by Goulet et al. [150], using biologging, found that female SES within the same foraging trip switch between different types of prey varying in their depth distribution, size, escape capacity, and bioluminescence, which are likely different species of myctophidae and, in lower proportions, squid species [89, 90]. These differences in foraging habitat and diet can cause a plastic behavioral response by the seals. For example, seals may adjust the number of prey they consume in response to the prey energy content [116] or seals may change their hunting mode in response to the size, the depth, and the anti-predator behavior of their prey [121].

In addition to among-individual variation in the plastic response to varying experienced environmental conditions, variation among individuals can emerge from intrinsic factors [151, 152]. For example, SES select distinct foraging habitats, varying in their productivity, level of competition and predation, and ice cover dynamics, with sex [66, 68], age [153], and temperament [78]. The diet of SES is mainly composed by fish and squid species [82, 84, 154, 155] and their relative proportions vary between individuals with sex [82] and age [86, 87]. Among-individual behavioral differences due to state variables, such as sex, age, body size, and temperament can be mediated by metabolic rate [156], energetical and nutritional needs [157], or diving capacity [146]. For example, the relationship between the diving metabolic limit of SES and swimming speed and dive duration varies among individuals [158], which may have direct consequences on the diving and hunting tactics they adopt while foraging.

The proportion of the variance explained by individual differences in the effect size between each metric and nPEE ($R_{S}^{2}$) was relatively stable at the scale of dives but varied substantially among metrics at the scale of days. $R_{S}^{2}$ was higher for dive-based metrics compared to track-based metrics. This variation among individuals in how they adjust their diving behavior resulted in predictions of nPEE with contrasting correlation values relative to the observed values. This suggests that the seals use different diving tactics. The bottom duration and the surface duration show the lowest values of $R_{F}^{2}$ and the highest variability among individuals in the direction of the metric’s effect size in response to nPEE. This result is interesting as it shows that there is no single dominant tactic among equipped seals in how they adjust the dive bottom phase duration and the post-dive surface duration in response to nPEE. For example, the following three tactics may exist according to the effect between surface duration and nPEE: a positive relationship may reflect individuals that increase surface time to recover from an increase in feeding effort [99]; an absence of relationship may indicate that seals adjust their diving behavior or metabolic rate to stabilize energy expenditure over time and avoid variation in surface duration [158–160]; and a negative relationship may be caused by seals reducing surface time in response to nPEE to the increase of feeding time while adopting alternative recovery tactics such as delayed recovery surface periods after intensive feeding bouts [161, 162] or during resting dives [163].

The GLMM that included the hunting time (computed from high-resolution dive profiles) resulted in extremely biased predictions for some individuals at the scale of dives and days (Fig. 5). Interestingly, this bias does not appear when fitting the model with BRT. After investigating the relationship between the hunting time and nPEE of these outlier individuals, we found that they have a non-linear relationship, which was accounted for by BRT. Therefore, individuals can vary in the direction of the effect size (positive or negative) between the metric and nPEE as well as in the shape of the relationship (linear or non-linear).

## Conclusion

In summary, our findings show that there is not a straightforward relationship between simple movement metrics and feeding intensity, which may be affected by several factors such as the temporal scale, individual variability, and the data resolution. We therefore conclude that these metrics should be carefully used, for example by testing them a priori with the studied species and the ecological context, and their limitations should be understood and taken into consideration. We also recommend computing most relevant metrics (e.g., ascent rate and hunting time in this study) from the raw high-resolution data even when only the summarized low-resolution data will be transmitted and accessible for researchers [e.g., 98]. For example, metrics could be computed onboard as the data is recorded and only their values transmitted through satellite communications.

Although considerable effort has been recently made to incorporate additional ecological complexity into foraging theoretical models [164, 165], its applicability remains still difficult and rare in field studies. More effort is thus needed to make modern methods of modeling foraging behavior more accessible to scientists, which will promote more effective wildlife management and conservation practices [166, 167].

## Supplementary Information

**Additional file 1. **General seal information.

**Additional file 2. **Building and checking the generalized linear mixed models.

**Additional file 3. **Example of hyperparameter tuning of boosted regression tree models.

**Additional file 4. **Inspecting the number of locations recorded per day.

**Additional file 5. **Output of all the generalized linear mixed-effect models.

## Data Availability

Datasets used for the models at the scale of dives and days are available on Zenodo: 10.5281/zenodo.7454415.

## Abbreviations

SES: Southern elephant seals
PEE: Prey encounter events
nPEE: Number of prey encounter events
ARS: Area-restricted search
FPT: First-passage time
GLMM: Generalized linear mixed-effect models
$R^{2}$: The coefficient of determination, the proportion of the total variance explained by the model
$R_{F}^{2}$: The proportion of the total variance explained by the predictors via the fixed slope variance
$R_{I}^{2}$: The proportion of the total variance explained by the individual-specific means via the random intercept variance
$R_{S}^{2}$: The proportion of the total variance explained by the predictors via the random slope variance/covariance
BRT: Boosted regression tree models
RMSE: Root-mean-square error
mRMSE: Mean-based normalized RMSE
sdRMSE: Standard-deviation-based normalized RMSE
