# Supplementary material for: Track and dive-based movement metrics do not predict the number of prey encountered by a marine predator
Source: Mov Ecol. 2023 Jan 21;11:3. doi: 10.1186/s40462-022-00361-2 (PMC9862577; doi:10.1186/s40462-022-00361-2)
Supplement: Supplementary file 1 — Additional file 1. General seal information. [file 40462_2022_361_MOESM1_ESM.pdf]

Additional file 1

General seal information

Allegue H., Réale D., Picard B., Guinet C. (2022) Track and dive-based movement metrics do not predict the number of prey encountered by a marine predator. *Mov. Ecol.*

Table S1: Descriptive information of the 65 equipped southern elephant seals including (Id) the seal identifier, (Date) the device deployment date, (Duration) the recording duration at sea in days, (Mass) the seal mass in kg, (Length) the seal body length from nose to tail in cm, (Location) whether locations were gps or argos quality, and the device type deployed on each seal individual: (SPLASH-10) SPLASH-10 logger, (TDR-ACC) time–depth recorder including an accelerometer, (TDR-FL-ACC) TDR-ACC including Fastloc GPS, and (CTD-SRDL) conductivity-temperature-depth-satellite relayed data logger (*includes accelerometer). The highlighted seals (n=21) are those that had complete recorded data for more than 30 consecutive days, which we retained for the analysis.

Id	Date	Duration (day)	Mass (kg)	Length (cm)	Location	SPLASH-10	TDR-ACC	TDR-FL-ACC	SPOT	CTD-SRDL
2010-18	2010-10-26	59	331.0	266	gps	x	x			
2010-19	2010-10-31	72	395.0	252	gps	x	x			
2010-21	2010-11-18	73	376.5	266	gps	x	x			
2011-14	2011-10-25	10	252.0	240	argos		x			x
2011-16	2011-10-26	9	255.0	254	gps	x	x			
2011-17	2011-10-26	9	225.0	225	argos		x			x
2011-18	2011-10-26	11	245.0	238	gps	x	x			
2011-21	2011-10-28	52	245.0	225	gps	x	x			
2011-26	2011-10-30	56	255.0	232	gps	x	x			
2011-27	2011-10-30	13	236.0	235	gps	x	x			
2011-28	2011-10-30	54	249.0	240	gps	x	x			
2012-1	2012-10-27	22	230.0	232	gps	x	x			
2012-11	2012-11-01	22	333.0	247	argos		x			x
2012-14	2012-11-01	27	258.0	229	argos		x			x
2012-15	2012-11-01	21	275.0	235	argos		x			x
2012-16	2012-11-01	24	425.0	265	argos		x			x
2012-17	2012-11-01	23	288.0	251	argos		x			x
2012-18	2012-11-02	24	328.0	252	argos		x			x
2012-2	2012-10-27	19	362.0	235	gps	x	x			
2012-3	2012-10-27	22	300.0	261	gps	x	x			
2012-4	2012-10-27	22	282.0	248	gps	x	x			
2012-6	2012-10-28	21	330.0	235	gps	x	x			
2012-9	2012-11-02	20	328.0	247	gps	x	x			
2013-1	2013-10-28	25	269.0	250	gps	x	x			
2013-10	2013-10-30	28	300.0	245	argos		x			x
2013-11	2013-10-30	26	277.0	240	argos		x			x
2013-12	2013-10-31	22	279.0	230	argos		x			x
2013-13	2013-10-31	22	264.0	240	argos		x			x
2013-16	2013-11-02	22	236.0	225	argos		x		x	
2013-18	2013-11-02	23	293.0	245	argos		x		x	
2013-2	2013-10-28	24	240.0	220	gps	x	x			
2013-3	2013-10-29	22	286.0	240	gps	x	x			

Table S1: *(Continued.)*

Id	Date	Duration (day)	Mass (kg)	Length (cm)	Location	SPLASH-10	TDR-ACC	TDR-FL-ACC	SPOT	CTD-SRDL
2013-4	2013-10-29	17	268.0	235	gps	x	x			
2013-5	2013-10-29	16	296.0	247	gps	x	x			
2013-6	2013-10-29	16	250.0	236	gps	x	x			
2013-7	2013-10-30	25	331.0	260	gps	x	x			
2014-22	2014-10-25	78	244.0	237	gps			x		
2014-23	2014-10-25	73	235.0	215	gps			x		
2014-24	2014-10-19	26	310.0	257	argos				x	x
2014-25	2014-10-22	17	350.0	269	gps	x	x			
2014-26	2014-10-22	16	285.0	232	gps	x	x			
2014-27	2014-10-23	17	230.0	232	gps	x	x			
2014-28	2014-10-20	15	214.5	219	gps	x	x			x
2014-29	2014-10-20	14	245.0	247	gps	x	x			x
2014-32	2014-10-26	17	242.0	222	argos		x		x	x
2014-33	2014-10-26	16	355.0	284	argos		x		x	x
2014-34	2014-10-17	16	236.5	231	argos		x			x
2014-35	2014-10-19	23	200.0	215	argos					x
2015-27	2015-10-29	73	223.4	259	gps			x		
2015-28	2015-10-30	71	283.0	243	argos		x		x	x
2015-30	2015-10-31	19	263.0	230	argos		x			x
2015-33	2015-11-02	21	292.0	250	argos		x			x
2015-34	2015-11-02	74	303.0	248	argos		x			x
2015-35	2015-11-02	19	241.0	232	argos		x			x
2015-36	2015-11-03	66	313.0	250	argos		x			x
2015-38	2015-11-03	17	282.0	232	argos		x			x
2018-30	2018-09-29	72	395.5	220	argos				x	x*
2018-37	2018-10-20	79	199.5	206	argos					x*
2018-38	2018-10-20	72	277.5	224	argos					x*
2018-39	2018-10-20	75	219.5	218	argos					x*
2018-40	2018-10-20	70	322.0	259	argos					x*
2018-41	2018-10-21	72	279.5	233	argos					x*
2019-14	2019-10-24	68	266.0	227	argos					x*
2019-2	2019-10-12	82	413.0	284	argos				x	x*
2019-3	2019-10-13	72	249.0	213	argos				x	x*
