# Supplementary material for: Track and dive-based movement metrics do not predict the number of prey encountered by a marine predator
Source: Mov Ecol. 2023 Jan 21;11:3. doi: 10.1186/s40462-022-00361-2 (PMC9862577; doi:10.1186/s40462-022-00361-2)
Supplement: Supplementary file 2 — Additional file 2. Building and checking the generalized linear mixed models. [file 40462_2022_361_MOESM2_ESM.pdf]

Additional file 2

Building and checking the generalized linear mixed models

Allegue H., Réale D., Picard B., Guinet C. (2022) Track and dive-based movement metrics do not predict the number of prey encountered by a marine predator. *Mov. Ecol.*

Contents

1 Introduction **1**

2 Model building at the scale of dives **2**

2.1 nPEE distribution 2

2.2 The starting model 3

2.3 Test and model overdispersion 4

2.4 Test and model zero-inflation 5

2.5 Test and model temporal autocorrelation 6

2.6 Compare the performance of all the models 6

3 Model checking at the scale of days **8**

3.1 nPEE distribution 8

3.2 The starting model 9

3.3 Test and model overdispersion 9

3.4 Test and model temporal autocorrelation 10

3.5 Compare the performance of all the models 10

4 Addtional residual violations **11**

References **11**

---

1 Introduction

In this document, we describe the model building and checking procedures we followed for the generalized linear mixed models (GLMM). The GLMM fit the number of prey encounter events (nPEE) as a function of the movement metrics at the scale of dives or days. We especially focus on investigating the presence of overdispersion, zero-inflation, and temporal autocorrelation. We also explain how we tackle each of these statistical aspects.

```r
# load libraries
library(data.table)
library(dplyr)

library(glmmTMB)
library(DHARMa)

set.seed(154)
```

2 Model building at the scale of dives

The data at the scale of dives is loaded and stored in the `dat` variable:

```r
dat
```

```
##         id_animal            datetime nPEE asc_rate_vs id_obs dive
##      1:   2010-18 2010-10-29 00:06:16    0   1.0463016      1    1
##      2:   2010-18 2010-10-29 00:24:19    0   0.9833386      2    2
##      3:   2010-18 2010-10-29 00:41:03    0   0.9509856      3    3
##      4:   2010-18 2010-10-29 00:59:48    0   0.8815842      4    4
##      5:   2010-18 2010-10-29 01:17:11    0   0.9122859      5    5
##     ---
## 100927:    2019-3 2019-12-31 22:00:05    3   0.9555556 100927 5726
## 100928:    2019-3 2019-12-31 22:22:50    1   1.0638888 100928 5727
## 100929:    2019-3 2019-12-31 22:49:16    6   1.1328571 100929 5728
## 100930:    2019-3 2019-12-31 23:15:47    7   1.1726914 100930 5729
## 100931:    2019-3 2019-12-31 23:42:09    0   1.3262500 100931 5730
```

- `id_animal`: is the seal unique identifier.
- `datetime`: is the date and time of the dive.
- `nPEE`: is the number of prey encounter events during the dive.
- `asc_rate_vs`: is the dive ascent rate.
- `id_obs`: is the observation unique identifier.
- `dive`: is the dive count for each seal.

2.1 nPEE distribution

In the Figure S1, we can see a large count of dives with no PEE. This suggests a possible zero-inflation in the data.

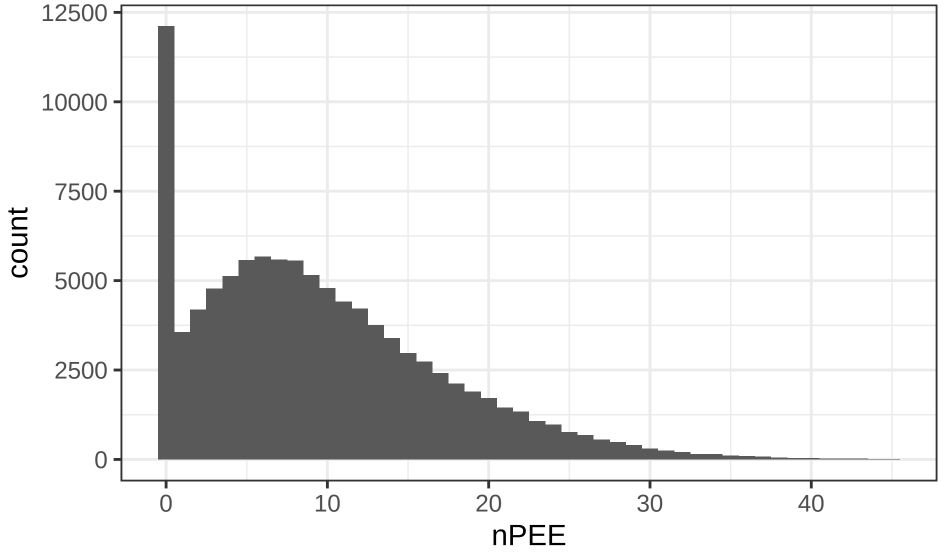

Figure S1: Histogram of nPEE per dive.

2.2 The starting model

The aim is to find the structure of the GLMM that will be used to model nPEE (i.e., count data) as a function of the movement metrics. Let's first start with fitting a model with a Poisson distribution and a log-link function (the *starting model*) as follow:

$$
\begin{aligned}
nPEE &\sim Poisson(\lambda_{ij}) \\
log(\lambda_{ij}) &= \beta_0 + u_{1,j} + \beta_1 x_{ij} + u_{2,j} x_{ij} + \epsilon_{ij}
\end{aligned}
$$

where λ_{ij} is the expected nPEE rate for the individual j at the dive i, x_{ij} is the movement metric value, β_0 is the population mean value, β_1 is the effect size of $log(\lambda_{ij})$ in response to x_{ij} (the slope), $u_{1,j}$ is the deviation from β_0 for individual j, $u_{2,j}$ is the deviation from β_1 for individual j, and ϵ_{ij} is the residual value.

$$
\begin{aligned}
\begin{bmatrix} u_1 \\ u_2 \end{bmatrix} &\sim \mathcal{N}\left(\boldsymbol{\mu_u} = \begin{bmatrix} 0 \\ 0 \end{bmatrix}, \boldsymbol{\Sigma_u} = \begin{bmatrix} \sigma^2_{u_1} & \sigma_{u_1}\sigma_{u_2} \\ \sigma_{u_1}\sigma_{u_2} & \sigma^2_{u_2} \end{bmatrix}\right) \\
\epsilon &\sim \mathcal{N}(0, \sigma^2_\epsilon)
\end{aligned}
$$

u_1 and u_2 follow a multivariate normal distribution with means of 0 and a covariance/variance matrix $\boldsymbol{\Sigma_u}$. $\sigma^2_{u_1}$ is the among-individual variance at the intercept, $\sigma^2_{u_2}$ is the among-individual variance at the slope with x, and $\sigma_{u_1}\sigma_{u_2}$ is the covariance between both. ϵ follows a normal distribution with a mean of 0 and variance of σ^2_ϵ.

Note that we use the dive ascent rate as predictor to run the model building procedure and apply the resulting model structure on all the GLMM. We use the `glmmTMB` R package to fit all models [1].

```
mods <- list() # a list that stores all candidate models

# the starting model
mods[["mod0"]] <- glmmTMB(
  nPEE ~ 1 + asc_rate_vs + (1+asc_rate_vs|id_animal),
```

```
  data      = dat,
  family    = "poisson"
)
```

2.3 Test and model overdispersion

We test the deviation of the dispersion of the starting model using the `DHARMa` R package. `DHARMa` uses a residual simulation-based approach to compare the model dispersion with the dispersion expected from simulations. We simulate residuals conditional to random effects (i.e., `re.form = NULL`). The output from the function `testDispersion()` shows that residuals are overdispersed ($V'_\epsilon > \sigma^2_\epsilon$).

```
DHARMa::testDispersion(
  DHARMa::simulateResiduals(
    mods[["mod0"]],
    re.form = NULL),
  plot=T
)
```

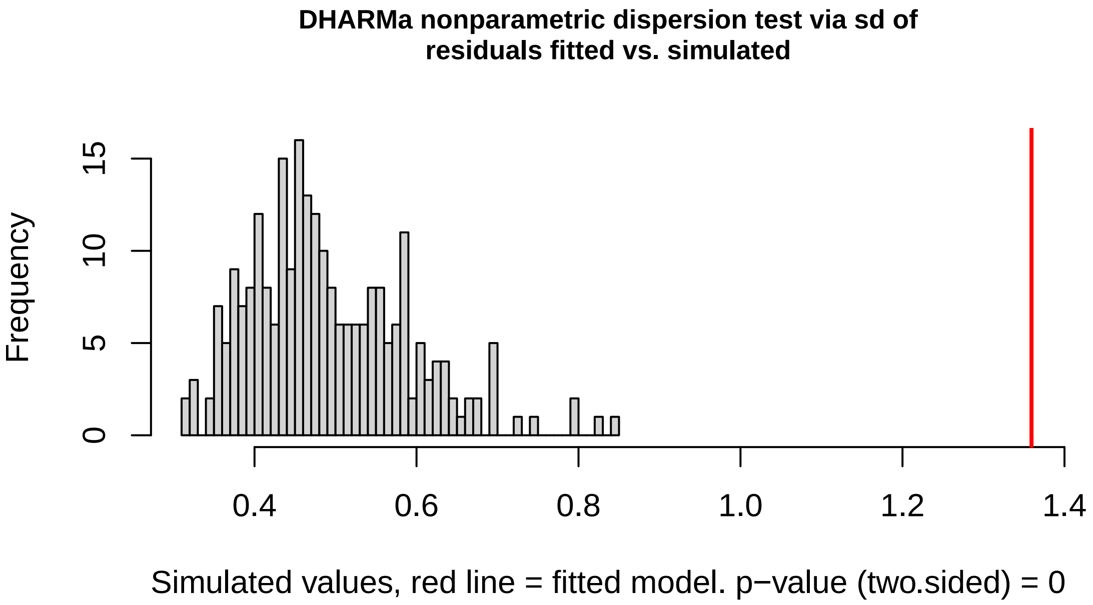


```
##
##  DHARMa nonparametric dispersion test via sd of residuals fitted vs.
##  simulated
##
## data:  simulationOutput
## dispersion = 2.7666, p-value < 2.2e-16
## alternative hypothesis: two.sided
```

To account for overdispersion, we add an observation-level random effect (OLRE) to the starting model [2]. This approach will also allow us to compute the relative contribution of each element of the model to the total variance, i.e., R^2_F, R^2_I, and R^2_S [3].

```r
mods[["mod.olre"]] <- update(
  mods[["mod0"]],
  formula = . ~ . + (1|id_obs)
)
```

2.4 Test and model zero-inflation

We test zero-inflation in the starting model using the `DHARMa` R package. `DHARMa` uses a simulation-based approach to compare the number of zeros observed with the number of zeros expected from simulations. The output of the function `testZeroInflation()` shows that the distribution of nPEE at the scale of dives is zero-inflated, i.e., more 0s than what expected from a Poisson distribution.

```r
DHARMa::testZeroInflation(
  DHARMa::simulateResiduals(
    mods[["mod0"]]),
  plot=T
)
```

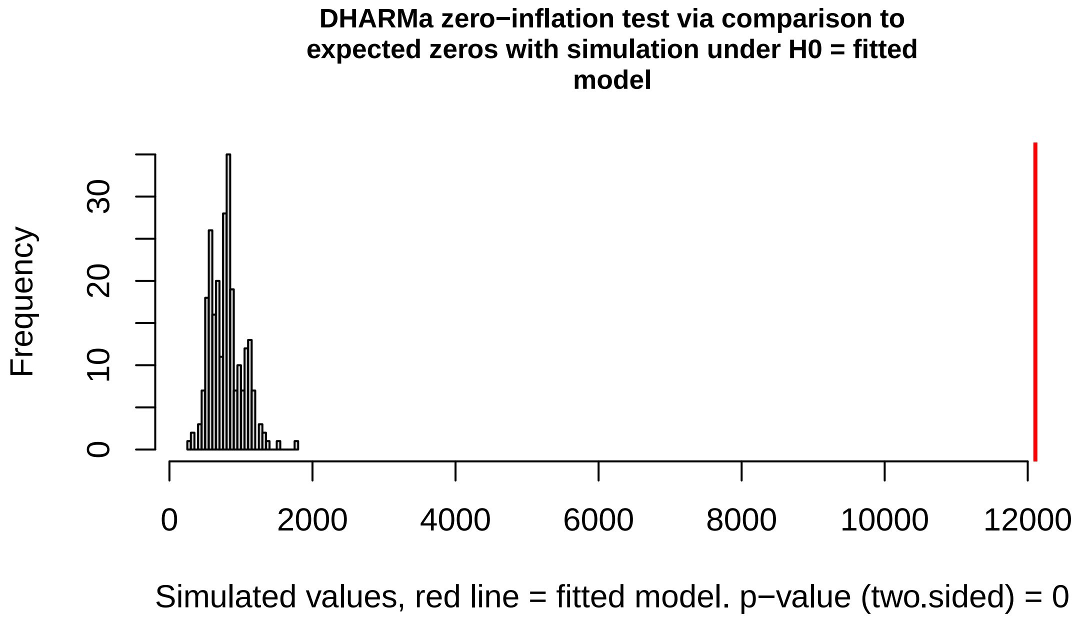


```
##
##  DHARMa zero-inflation test via comparison to expected zeros with
##  simulation under H0 = fitted model
##
## data:  simulationOutput
## ratioObsSim = 15.2, p-value < 2.2e-16
## alternative hypothesis: two.sided
```

To account for zero-inflation, we fit a zero-inflated model that assumes the data comes from a mixture of two generative processes:

1. The first underlying process determines whether nPEE is zero (no feeding mode, e.g., transiting) or not (feeding mode). This process is represented by a binomial model that models the probability a dive has no PEE.

2. When the seal is considered in a feeding mode, a second process takes over to model nPEE which may also results in nPEE=0 if the seal does not encounter any prey. This process can be modeled by a Poisson distribution.

```
mods[["mod.zi"]] <- update(
  mods[["mod0"]],
  ziformula = ~ 1
)
```

2.5 Test and model temporal autocorrelation

To investigate autocorrelation in nPEE, we plot the correlation values of the residuals for different dive lags. For a dive lag of 1, 2, 3, . . . , n, the correlation is computed between the current dive and, respectively, the 1st, 2nd, 3rd, . . . , n^{th} preceding dive. We plot distinct curves for each of the seals as well as the mean autocorrelation curve in red. The figure S2 shows that there is substantial correlation at a lag of 1 dive. This correlation decreases as the dive lag increases and seems to stabilize around a lag of 10 dives. Autocorrelation values at a lag of 1 dive vary among individuals ranging approximately from 0.25 to 0.65.

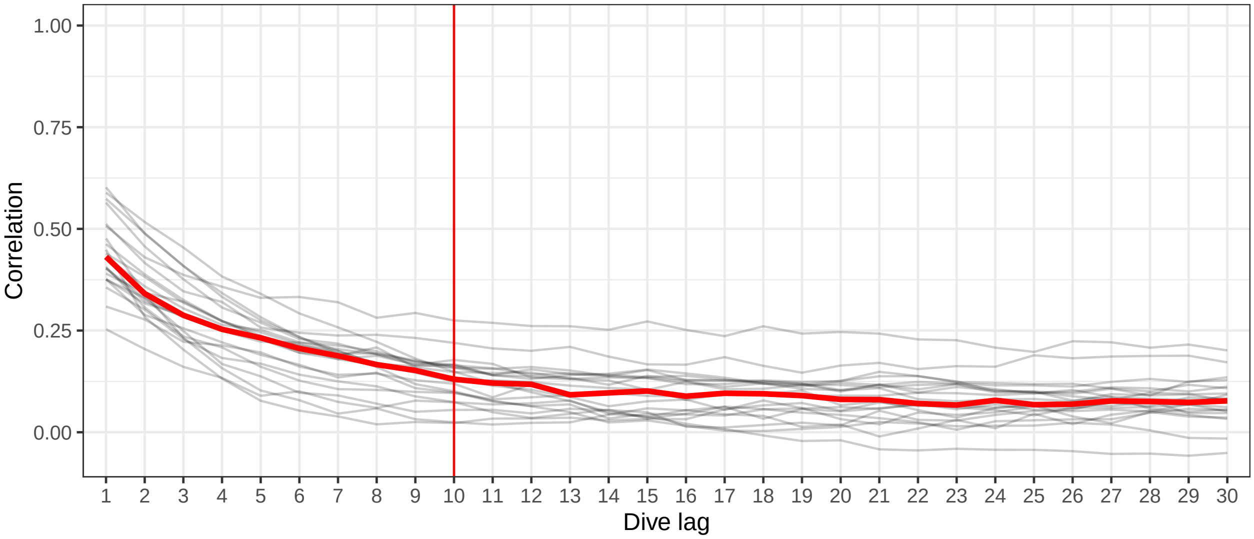

Figure S2: Autocorrelation function plot of the simulated residuals of the starting model (mod0) at the scale of dives. Each line represents an individual seal and the red line is the mean value.

To account for the autocorrelation, we add to the model a (co)variance matrix with an autoregressive process of order 1.

```
mods[["mod.autocor"]] <- update(
  mods[["mod0"]],
  formula =  . ~ . + ar1(0+dive|id_animal)
)
```

2.6 Compare the performance of all the models

As the goal of this study is to compare the performance of different movement metrics in predicting nPEE, we test if accounting for overdispersion, zero-inflation, and autocorrelation improves the predictive performance

of the starting model. The function `run_CV_RMSE()` splits the data into two datasets: 60% of the data to train the model and 40% to test it. Then `run_CV_RMSE()` computes and returns the root mean square error (RMSE) and the correlation value between the predicted values and the observed values of the testing dataset. we run `run_CV_RMSE()` for each of the models and present the results in Table S1.

```
run_CV_RMSE <- function(mod){

  # partition the data (within each seal) into
  # training (60%) and testing (40%) datasets
  dat[ , train := sample(c(TRUE, FALSE), .N,
                         replace=T,
                         prob=c(0.6, 0.4)),
      by=id_animal]

  # create training and testing datasets
  train <- dat[train == TRUE]
  test  <- dat[train == FALSE]

  # refit the model with the training dataset
  # and predict the testing dataset
  pred <- update(mod, data = train) %>%
          predict(newdata          = test,
                  re.form          =  NA,
                  allow.new.levels = TRUE,
                  type             = "response")

  # compute RMSE and COR
  data.frame(
    "RMSE"   = caret::RMSE(pred = pred, obs  = test[["nPEE"]]),
    "COR"    = cor(pred, test[["nPEE"]])
  )
}

# get and combine the results together
dt_res <- do.call(rbind, lapply(mods, run_CV_RMSE))
```

Table S1: Comparison of the predictive capacity of all the models at the scale of dives.

	RMSE	COR
mod0	7.036686	0.3954438
mod.olre	7.524799	0.3688857
mod.zi	6.975116	0.4150810
mod.autocor	7.309238	0.4003287

RMSE is the root mean square error and COR is the correlation value between the predicted values and the observed values.

We can see that the performance of the starting model is not improved when accounting for overdispersion, zero-inflation, and autocorrelation. Therefore, to keep the model structure as simple as possible, we decide to keep the starting model to which we add an observation level random effect as we need it to decompose the different variance components of the model.

3 Model checking at the scale of days

We repeat the same steps described above to conduct model checking on the GLMM at the scale of days. The data at the scale of days is loaded and stored in the `dat` variable.

```
dat
```

```
##        id_animal   datetime nPEE asc_rate_vs id_obs day
##    1:    2010-18 2010-10-29   23   0.9203202      1   1
##    2:    2010-18 2010-10-30  391   1.1247575      2   2
##    3:    2010-18 2010-10-31  500   1.2655546      3   3
##    4:    2010-18 2010-11-01  444   1.2845490      4   4
##    5:    2010-18 2010-11-02  394   1.2665724      5   5
##   ---
## 1459:     2019-3 2019-12-27  540   1.1978099   1459  68
## 1460:     2019-3 2019-12-28  414   1.1372323   1460  69
## 1461:     2019-3 2019-12-29  265   1.2158821   1461  70
## 1462:     2019-3 2019-12-30  195   1.1325307   1462  71
## 1463:     2019-3 2019-12-31  298   1.1351892   1463  72
```

- `id_animal`: is the seal unique identifier.
- `datetime`: is the date.
- `nPEE`: is the number of prey encounter events during the day
- `asc_rate_vs`: is the average dive ascent rate.
- `id_obs`: is the observation unique identifier.
- `day`: is the day count for each seal.

3.1 nPEE distribution

As nPEE is always higher than 0 for all days of all seals, we will not test for zero-inflation.

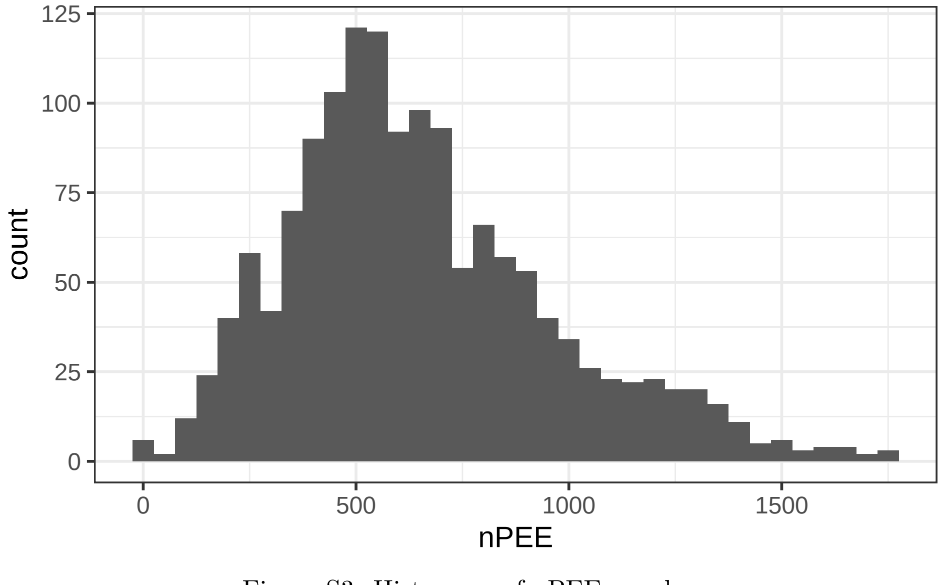

Figure S3: Histogram of nPEE per day.

3.2 The starting model

```
mods <- list() # a list that stores all candidate models

# the starting model
mods[["mod0"]] <- glmmTMB(
  nPEE ~ 1 + asc_rate_vs + (1 + asc_rate_vs|id_animal),
  data      = dat,
  family    = "poisson"
)
```

3.3 Test and model overdispersion

The output from the function `testDispersion()` does not show any significant dispersion of the residuals.

```
DHARMa::testDispersion(
  DHARMa::simulateResiduals(
    mods[["mod0"]],
    re.form = NULL),
  plot=T
)
```

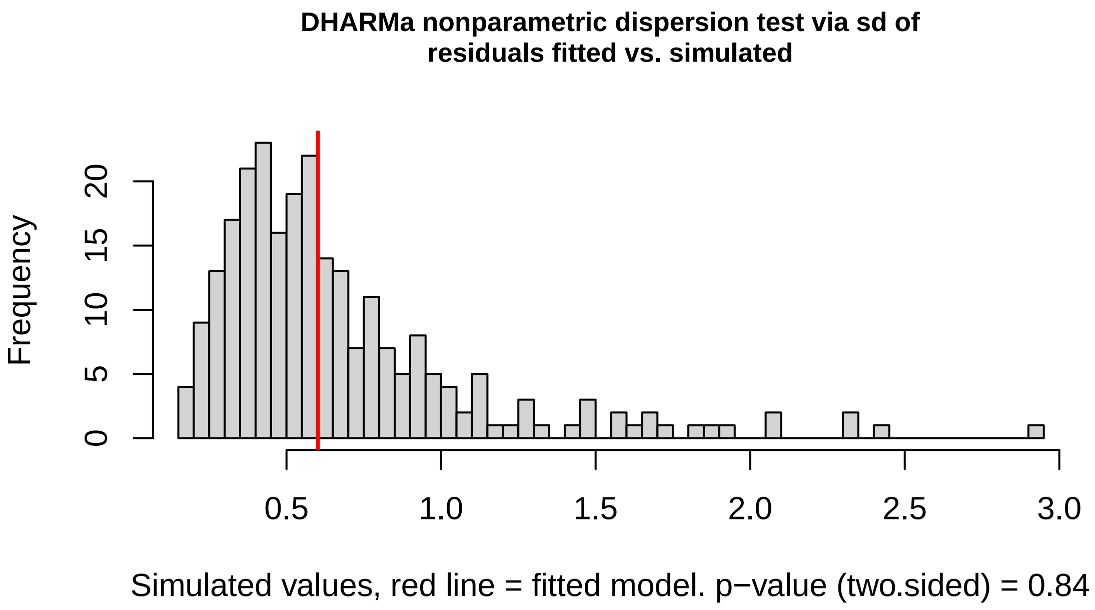


```
##
##  DHARMa nonparametric dispersion test via sd of residuals fitted vs.
##  simulated
##
## data:  simulationOutput
## dispersion = 0.8929, p-value = 0.84
## alternative hypothesis: two.sided
```

3.4 Test and model temporal autocorrelation

The figure S4 shows that there is substantial correlation at a lag of 1 day. This correlation decreases as the day lag increases and seems to stabilize around a lag of 7 days. Autocorrelation values at a lag of 1 day vary among individuals ranging approximately from 0.2 to 0.8.

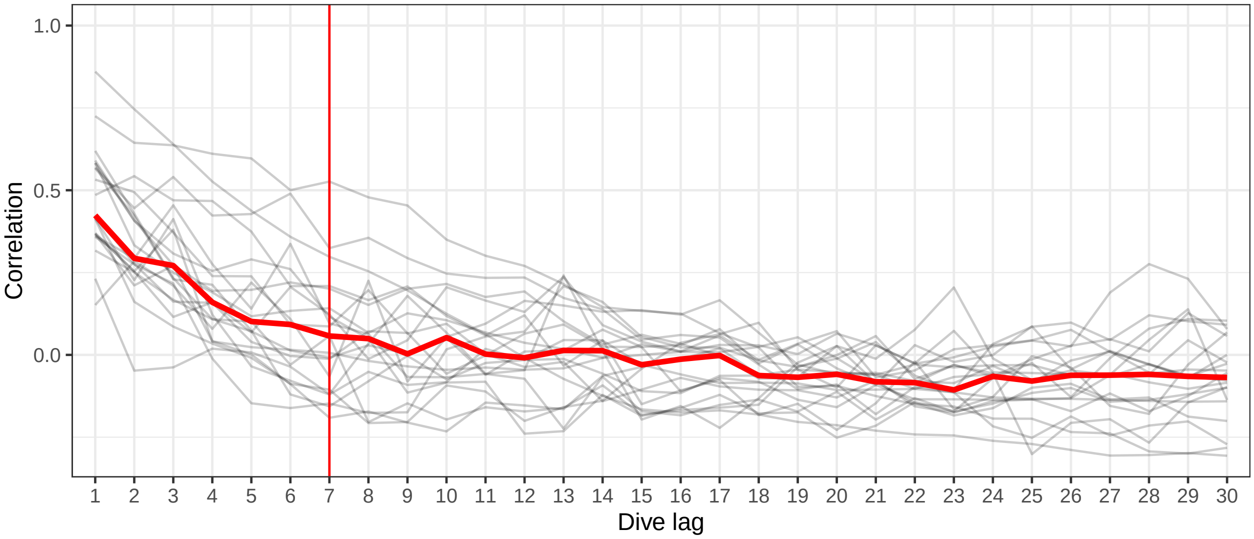

Figure S4: Autocorrelation function plot of the simulated residuals of the starting model (mod0) at the scale of days. Each line represents an individual seal and the red line is the mean value.

To account for the autocorrelation, we add to the model a (co)variance matrix with an autoregressive process of order 1.

```
mods[["mod.autocor"]] <- update(
  mods[["mod0"]],
  formula =  . ~ . + ar1(0+day|id_animal)
)
```

3.5 Compare the performance of all the models

we run `run_CV_RMSE()` for each of the models and present the results in Table S2.

```
# get and combine the results together
dt_res <- do.call(rbind, lapply(mods, run_CV_RMSE))
```

Table S2: Comparison the predictive capacity of all the models at the scale of dives.

	RMSE	COR
mod0	340.3596	0.2178858
mod.autocor	341.0145	0.2700253

RMSE is the root mean square error and COR is the correlation value between the predicted values and the observed values.

We can see that the performance of the starting model is not improved when accounting for autocorrelation. Similarly to the scale of dives, we keep the starting model to which we add an observation level random effect as we need it to decompose the different variance components of the model.

4 Addtional residual violations

Additional violations in the model residuals are likely due to missing predictors/interactions or nonlinear relationships. We are not going to consider these additional residual violations in our model checking procedure as our goal is to test the performance of the movement metrics alone in predicting nPEE. The interactions between predictors and the non-linearity of relationships are implicitly tackled by the boosted regression tree models.

References

1. Brooks ME, Kristensen K, Benthem KJ van, Magnusson A, Berg CW, Nielsen A, et al. glmmTMB balances speed and flexibility among packages for zero-inflated generalized linear mixed modeling. R J. Technische Universitaet Wien; 2017;9:378–400.
2. Harrison XA. Using observation-level random effects to model overdispersion in count data in ecology and evolution. Miao C, editor. PeerJ [Internet]. 2014;2:e616. Available from: https://doi.org/10.7717/peerj.616
3. Nakagawa S, Schielzeth H. A general and simple method for obtaining R2 from generalized linear mixed-effects models. Methods Ecol Evol. 2013;4:133–42.
