# Supplementary material for: Track and dive-based movement metrics do not predict the number of prey encountered by a marine predator
Source: Mov Ecol. 2023 Jan 21;11:3. doi: 10.1186/s40462-022-00361-2 (PMC9862577; doi:10.1186/s40462-022-00361-2)
Supplement: Supplementary file 3 — Additional file 3. Example of hyperparameter tuning of boosted regression tree models. [file 40462_2022_361_MOESM3_ESM.pdf]

Additional file 3

Example of hyperparameter tuning of the boosted regression tree models

Allegue H., Réale D., Picard B., Guinet C. (2022) Track and dive-based movement metrics do not predict the number of prey encountered by a marine predator. *Mov. Ecol.*

Contents

1 Introduction **2**

2 Prepare training and testing datasets **2**

3 Hyperparameter tuning steps **3**

3.1 Default hyperparameter values . . . 3

3.2 Set initial parameter values . . . 3

3.3 Best number of iterations (trees) . . . 4

3.4 Prepare hyperparameter tuning . . . 4

3.5 Tune `tweedie_variance_power` . . . 5

3.6 Tune `max_depth` and `min_child_weight` . . . 6

3.7 Tune `gamma` . . . 6

3.8 Tune `subsample` . . . 6

3.9 Tune the regularization parameter `alpha` . . . 7

3.10 Fit the tuned model . . . 7

3.11 Compare tuned vs default models . . . 8

4 Summary: hyperparameter initial values **8**

5 Summary: hyperparameter tuning space **9**

6 Summary: tuned hyperparameter values **9**

6.1 At the scale of dives . . . 9

6.2 At the scale of days . . . 11

References **12**

---

1 Introduction

In this document, we present an example of the steps we followed to tune the hyperparameters of the boosted regression tree (BRT) models. In this example, we model the number of prey encounter events (nPEE) as a function of the mean dive ascent rate at the scale of days.

```
# load libraries
library(data.table)
library(dplyr)

library(xgboost) # for fitting the xgboost model
library(caret)   # tools for building machine learning models
library(mlr)     # for hyperparameter tuning

library(parallel)
library(parallelMap)

set.seed(514)
```

The data is loaded and stored in the `dat` variable:

```
head(dat)
```

```
##    id_animal nPEE  asc_rate
## 1:   2010-18   23 0.9203202
## 2:   2010-18  391 1.1247575
## 3:   2010-18  500 1.2655546
## 4:   2010-18  444 1.2845490
## 5:   2010-18  394 1.2665724
## 6:   2010-18  407 1.3100665
```

- `id_animal`: is the seal unique identifier.
- `nPEE`: is the number of prey encounter events.
- `asc_rate`: is the mean dive ascent rate.

Details on how `asc_rate` is calculated is presented in the *Methods* section of the main document. The dive ascent rate metric used in this protocol is computed from the high-resolution dive data (1 Hz).

2 Prepare training and testing datasets

We use 60% of the dataset to train and the other 40% to test the model.

```
# save variable names
target   <- "nPEE"     # dependent variable
features <- "asc_rate" # independent variable
vars     <- c(target, features)

# partition the data (within each seal) into training (60%) and testing (40%) datasets
dat[ , train := sample(c(TRUE, FALSE), .N, replace=T, prob=c(0.6, 0.4)), by=id_animal]

train <- dat[train == TRUE]
test  <- dat[train == FALSE]
```

3 Hyperparameter tuning steps

We use the following steps for tuning the model hyperparameters:

3.1 Default hyperparameter values

We fit a model with the default hyperparameter values and compute its accuracy (root-mean-square error; RMSE). We use the XGBoost system, a scalable machine learning system for tree boosting [1], to fit the model. We use a tree-based booster with the tweedie distribution as objective to model nPEE. The tweedie distribution is suitable for our dataset as it models dispersion and accounts for zero-inflation [2].

```
# prepare training and testing matrix
xgb_train <- xgb.DMatrix(
  data  = data.matrix(train[ , ..features]),
  label = train[[target]]
)
xgb_test  <- xgb.DMatrix(
  data  = data.matrix(test[ , ..features]),
  label = test[[target]]
)

# set general parameters
params <- list(
  booster     = "gbtree",
  objective   = "reg:tweedie"
)

#train model
xgb_default <-  xgb.train(
  params   = params,
  nrounds  = 100L,
  data     = xgb_train,
  verbose  = 0
)

# compute accuracy of the default training model
RMSE_default <- caret::RMSE(
  pred = predict(xgb_default, xgb_test),
  obs  = test[[target]]
)
```

3.2 Set initial parameter values

We set initial values for the following XGBoost hyperparameters:

- `tweedie_variance_power`: parameter that controls the variance of the tweedie distribution (default=1.5, range=[1,2]).
- `eta`: step size shrinkage or learning rate (default=0.3, range=[0,1])
- `max_depth`: maximum tree depth (default=6).
- `min_child_weight`: minimum sum of instance weight needed in a child (default=1, range=[0,∞]).
- `gamma`: minimum loss reduction required to make a further partition on a leaf node of the tree (default=0, range=[0,∞]).

- `subsample`: subsample ratio of the training instances (default=1, range=(0,1]).
- `alpha`: L1 regularization term on weights (default=0, range=[0,∞]).

For more information about XGBoost hyperparameters, see the official website: https://xgboost.readthedocs.io/en/latest/parameter.html

```
# initial parameters
params <- list(
  booster                = "gbtree",
  objective              = "reg:tweedie",
  tree_method            = "hist",
  tweedie_variance_power = 1.5,
  eta                    = 0.1,
  max_depth              = 3,
  min_child_weight       = 50,
  gamma                  = 0,
  subsample              = 0.9,
  alpha                  = 0
)
```

3.3 Best number of iterations (trees)

From a 5-fold cross-validation, we determine the best number of iterations needed by the model fitted with the initial hyperparameter values. The model will stop if its accuracy is not improved after 10 iterations.

```
# compute cross-validation
# to find the best number of iterations
xgb_cv <- xgb.cv(
  params                = params,
  data                  = xgb_train,
  nrounds               = 500L,
  nfold                 = 5,
  early_stopping_rounds = 10,
  maximize              = FALSE,
  verbose               = 0)

# best number of iterations
(nrounds <- xgb_cv$best_iteration)
```

```
## [1] 69
```

```
# update parameter list
params[["nrounds"]] <- nrounds
```

3.4 Prepare hyperparameter tuning

```
#create training task
train_task <- makeRegrTask(
  data   = as.data.frame(train[ , ..vars]),
  target = target
```

```
)

# function for hyperparameter tuning
tune_parameters <- function(p, p_space){

  ## p: the list of the model parameters
  ## p_space: an object defining all the parameter
  ##          combination values to be tested

  # create learner
  xgb_learner <- makeLearner("regr.xgboost", par.vals = p)

  # search strategy
  control <- makeTuneControlGrid()

  # set resampling strategy
  resample_desc <- makeResampleDesc("CV", iters = 4L)

  set.seed(123)

  # hyperparameter tuning
  tuned_p <- tuneParams(
    learner    = xgb_learner,
    task       = train_task,
    resampling = resample_desc,
    par.set    = p_space,
    control    = control,
    show.info  = TRUE
  )

  return(tuned_p$x)
}
```

3.5 Tune tweedie_variance_power

```
# set hyperparameter tuning space
params_space <- makeParamSet(
  makeDiscreteParam("tweedie_variance_power", values = seq(1.2, 1.8, .15))
)

# tune parameters
tuned_params <- tune_parameters(params, params_space)

# best tweedie_variance_power value
(tweedie_variance_power <- tuned_params$tweedie_variance_power)
```

```
## [1] 1.5
```

```
# update parameter list
params[["tweedie_variance_power"]] <- tweedie_variance_power
```

3.6 Tune max_depth and min_child_weight

```
# set hyperparameter tuning space
params_space <- makeParamSet(
  makeDiscreteParam("max_depth",        values = c(2, 3, 4, 6, 8)),
  makeDiscreteParam("min_child_weight", values = c(1, 10, 25, 50, 100, 200,
                                                   300, 400, 500, 750, 1000))
)

# tune parameters
tuned_params <- tune_parameters(params, params_space)

# best max_depth and min_child_weight values
(max_depth       <- tuned_params$max_depth)
```

```
## [1] 2
```

```
(min_child_weight<- tuned_params$min_child_weight)
```

```
## [1] 10
```

```
# update parameter list
params[["max_depth"]]        <- max_depth
params[["min_child_weight"]] <- min_child_weight
```

3.7 Tune gamma

```
# set hyperparameter tuning space
params_space <- makeParamSet(
  makeDiscreteParam("gamma", values = c(0, 10, 20, 30, 40, 50, 60))
)

# tune parameters
tuned_params <- tune_parameters(params, params_space)

# best gamma value
(gamma <- tuned_params$gamma)
```

```
## [1] 30
```

```
# update parameter list
params[["gamma"]] <- gamma
```

3.8 Tune subsample

```r
# set hyperparameter tuning space
params_space <- makeParamSet(
  makeDiscreteParam("subsample", values = seq(0.6, 1, .2))
)

# tune parameters
tuned_params <- tune_parameters(params, params_space)

# best subsample
(subsample <- tuned_params$subsample)
```

```
## [1] 0.8
```

```r
# update parameter list
params[["subsample"]] <- subsample
```

3.9 Tune the regularization parameter alpha

```r
# set hyperparameter tuning space
params_space <- makeParamSet(
  makeDiscreteParam("alpha", values = c(0, 3, 5, 7.5, 10, 25, 50,
                                        75, 100, 125, 150, 200))
)

# tune parameters
tuned_params <- tune_parameters(params, params_space)

# best alpha
(alpha <- tuned_params$alpha)
```

```
## [1] 5
```

```r
# update parameter list
params[["alpha"]] <- alpha
```

3.10 Fit the tuned model

```r
#train model
xgb_tuned <-  xgb.train(
  # remove nrounds from the parameter list
  params    = params[names(params)[which(names(params) != "nrounds")]],
  data      = xgb_train,
  nrounds   = params[["nrounds"]],
  verbose   = 0
)

# compute accuracy of the tuned training model
```

```r
RMSE_tuned <- caret::RMSE(
  pred = predict(xgb_tuned, xgb_test),
  obs  = test[[target]]
)
```

3.11 Compare tuned vs default models

Table S1: Hyperparameter values of the tuned model and the accuracy improvement relative to the default model.

Parameter	Value
`nrounds`	69
`tweedie_variance_power`	1.50
`eta`	0.1
`max_depth`	2
`min_child_weight`	10
`gamma`	30
`subsample`	0.8
`alpha`	5
RMSE (default)	352.8
RMSE (tuned)	299.7
%ΔRMSE	15.0%

We use the root-mean-square error (RMSE) to quantify the accuracy of the models. %ΔRMSE is percentage of improvement of the RMSE of the model fitted with the tuned hyperparameters compared to the RMSE of the model fitted with the default hyperparameters.

4 Summary: hyperparameter initial values

Table S2: Initial hyperparameter values used for the BRT models at the scale of dives and days.

	At the scale of	
Parameter	dives	days
`tweedie_variance_power`	1.5	1.5
`eta`	0.3	0.1
`max_depth`	3	3
`min_child_weight`	500	50
`gamma`	0	0
`subsample`	0.9	0.9
`alpha`	0	0

5 Summary: hyperparameter tuning space

Table S3: The hyperparameter tuning space used for the BRT models at the scale of dives and days.

	At the scale of	
Parameter	dives	days
`tweedie_variance_power`	1.20; 1.35; 1.50; 1.65; 1.80	1.20; 1.35; 1.50; 1.65; 1.80
`eta`	2; 4; 6; 8	2; 3; 4; 6; 8
`max_depth`	1; 25; 50; 100; 300; 600; 900; 1200; 1500; 2000; 2500; 3000	1; 10; 25; 50; 100; 200; 300; 400; 500; 750; 1000
`min_child_weight`	0; 2.5; 5; 7.5; 10; 12.5; 15; 20	0; 10; 20; 30; 40; 50; 60
`gamma`	0.6; 0.8; 1.0	0.6; 0.8; 1.0
`subsample`	0; 3; 5; 7.5; 10; 25; 50; 75; 100; 125; 150; 200	0; 3; 5; 7.5; 10; 25; 50; 75; 100; 125; 150; 200
`alpha`	1.20; 1.35; 1.50; 1.65; 1.80	1.20; 1.35; 1.50; 1.65; 1.80

6 Summary: tuned hyperparameter values

We present here a summary of the tuned hyperparameter values of all the models at the scale of dives and days.

6.1 At the scale of dives

Table S4: Tuned hyperparameter values of the BRT models at the scale of dives and include one of the dive-based metrics computed from the high-resolution dive data.

Parameter	Desc. rate	Asc. rate	Bottom dur.	Surface dur.	Efficiency	Sinuosity	Hunt. time
`nrounds`	20	25	19	150	20	23	26
`tweedie_variance_power`	1.65	1.35	1.50	1.20	1.50	1.50	1.20
`eta`	0.3	0.3	0.3	0.3	0.3	0.3	0.3
`max_depth`	2	2	2	6	2	2	6
`min_child_weight`	1200	100	1200	3000	100	50	25
`gamma`	0	10	0	0	0	2	0
`subsample`	0.8	0.6	1.0	1.0	0.6	0.6	1.0
`alpha`	5	5	3	3	0	25	0
RMSE (default)	7.16	6.88	7.37	7.39	7.26	7.28	7.06
RMSE (tuned)	7.13	6.86	7.35	7.38	7.20	7.25	7.11
%ΔRMSE	0.47%	0.36%	0.26%	0.10%	0.86%	0.37%	-0.75%

Table S5: Tuned hyperparameter values of the BRT models at the scale of dives and include one of the dive-based metrics computed from the low-resolution dive data.

Parameter	Desc. rate	Asc. rate	Bottom dur.	Efficiency	Hunt. time
nrounds	20	20	20	19	22
tweedie_variance_power	1.35	1.65	1.65	1.50	1.50
eta	0.3	0.3	0.3	0.3	0.3
max_depth	2	2	2	2	2
min_child_weight	100	300	25	25	50
gamma	10	2	2	2	2
subsample	0.8	0.6	0.6	1.0	1.0
alpha	3	0	50	10	8
RMSE (default)	7.46	7.42	7.38	7.44	7.26
RMSE (tuned)	7.41	7.37	7.38	7.37	7.24
%ΔRMSE	0.67%	0.67%	0.02%	0.96%	0.27%

Table S6: Tuned hyperparameter values of the BRT models at the scale of dives and include one of the track-based metrics.

Parameter	H. speed	Turn. angle	FPT	Move pers.
nrounds	17	17	35	127
tweedie_variance_power	1.35	1.35	1.65	1.35
eta	0.3	0.3	0.3	0.3
max_depth	2	2	4	4
min_child_weight	50	50	25	1200
gamma	0	10	2	0
subsample	0.8	1.0	0.8	1.0
alpha	50	3	3	0
RMSE (default)	7.64	7.78	7.52	7.54
RMSE (tuned)	7.60	7.72	7.54	7.54
%ΔRMSE	0.56%	0.67%	-0.26%	0.00%

Table S7: Tuned hyperparameter values of the BRT models at the scale of dives and include all the dive-based metrics (Dive), all the track-based metrics (Track), or all the metrics (All).

Parameter	Dive	Track	All
nrounds	171	155	266
tweedie_variance_power	1.20	1.20	1.20
eta	0.3	0.3	0.3
max_depth	4	6	6
min_child_weight	100	100	900
gamma	2	0	0
subsample	1.0	1.0	1.0
alpha	5	25	125
RMSE (default)	5.47	7.14	5.33
RMSE (tuned)	5.47	7.03	5.18
%ΔRMSE	-0.06%	1.51%	2.67%

6.2 At the scale of days

Table S8: Tuned hyperparameter values of the BRT models at the scale of days and include one of the dive-based metrics computed from the high-resolution dive data.

Parameter	Desc. rate	Asc. rate	Bottom dur.	Surface dur.	Efficiency	Sinuosity	Hunt. time
`nrounds`	69	71	68	71	71	78	73
`tweedie_variance_power`	1.50	1.50	1.50	1.50	1.50	1.65	1.50
`eta`	0.1	0.1	0.1	0.1	0.1	0.1	0.1
`max_depth`	2	2	2	2	2	2	2
`min_child_weight`	1	25	1000	400	300	300	200
`gamma`	20	30	20	30	20	10	20
`subsample`	0.6	0.6	0.6	0.6	0.6	0.8	0.6
`alpha`	0	10	0	10	3	0	0
RMSE (default)	359.4	356.9	373.2	351.8	319.7	374.0	389.6
RMSE (tuned)	298.0	293.2	307.2	298.9	275.8	310.6	305.5
%ΔRMSE	17.1%	17.8%	17.7%	15.0%	13.7%	17.0%	21.6%

Table S9: Tuned hyperparameter values of the BRT models at the scale of days and include one of the dive-based metrics computed from the low-resolution dive data.

Parameter	Desc. rate	Asc. rate	Bottom dur.	Efficiency	Hunt. time
`nrounds`	74	68	68	66	68
`tweedie_variance_power`	1.50	1.50	1.50	1.50	1.50
`eta`	0.1	0.1	0.1	0.1	0.1
`max_depth`	2	2	2	2	2
`min_child_weight`	10	300	1000	50	50
`gamma`	10	10	20	20	20
`subsample`	0.6	0.8	0.6	0.6	0.6
`alpha`	0	5	3	5	10
RMSE (default)	395.4	368.0	382.9	344.6	378.9
RMSE (tuned)	306.3	309.3	308.7	283.8	308.2
%ΔRMSE	22.5%	15.9%	19.4%	17.7%	18.7%

Table S10: Tuned hyperparameter values of the BRT models at the scale of days and include one of the track-based metrics.

Parameter	H. speed	Turn. angle	FPT	Move pers.
`nrounds`	67	71	71	69
`tweedie_variance_power`	1.50	1.50	1.50	1.50
`eta`	0.1	0.1	0.1	0.1
`max_depth`	3	2	2	2
`min_child_weight`	1000	1000	50	50
`gamma`	30	0	10	20
`subsample`	0.6	0.8	0.8	0.8
`alpha`	10	3	0	5
RMSE (default)	364.2	393.3	357.0	362.4
RMSE (tuned)	306.1	315.0	297.1	290.8
%ΔRMSE	15.9%	19.9%	16.8%	19.8%

Table S11: Tuned hyperparameter values of the BRT models at the scale of days and include all the dive-based metrics (Dive), all the track-based metrics (Track), or all the metrics (All).

Parameter	Dive	Track	All
`nrounds`	150	86	164
`tweedie_variance_power`	1.50	1.65	1.35
`eta`	0.1	0.1	0.1
`max_depth`	8	4	8
`min_child_weight`	25	200	400
`gamma`	0	0	0
`subsample`	0.6	1.0	0.8
`alpha`	0	0	25
RMSE (default)	146.3	288.0	156.7
RMSE (tuned)	136.1	260.3	147.8
%ΔRMSE	7.0%	9.6%	5.7%

References

1. Chen T, Guestrin C. XGBoost: A scalable tree boosting system. Proc ACM SIGKDD Int Conf Knowl Discov Data Min [Internet]. Association for Computing Machinery; 2016;13-17-Augu:785–94. Available from: https://arxiv.org/abs/1603.02754

2. Zhou H, Qian W, Yang Y. Tweedie gradient boosting for extremely unbalanced zero-inflated data. Commun Stat - Simul Comput. Taylor & Francis; 2020;1–23.
