# Supplementary material for: Track and dive-based movement metrics do not predict the number of prey encountered by a marine predator
Source: Mov Ecol. 2023 Jan 21;11:3. doi: 10.1186/s40462-022-00361-2 (PMC9862577; doi:10.1186/s40462-022-00361-2)
Supplement: Supplementary file 4 — Additional file 4. Inspecting the number of locations recorded per day. [file 40462_2022_361_MOESM4_ESM.pdf]

Additional file 4

Inspecting the number of locations recorded per day

Allegue H., Réale D., Picard B., Guinet C. (2022) Track and dive-based movement metrics do not predict the number of prey encountered by a marine predator. *Mov. Ecol.*

When inspecting the quality of the data for our analysis, we noticed that some seals (n=2) had a low sampling rate of recorded locations along their tracks. To avoid any potential bias in our models due to this low sampling rate in locations, we decided to exclude these individuals from all models that involved track-based metrics. The histogram of the mean number of locations per day for all the seals shows that the problematic seals can be excluded by a threshold of <15 location/day (Figure @ref(fig:histnlocs)). The Figure @ref(fig:mapnlocs) and @ref(fig:nlocsday) show an example of a seal that was retained and one that was excluded from the analysis.

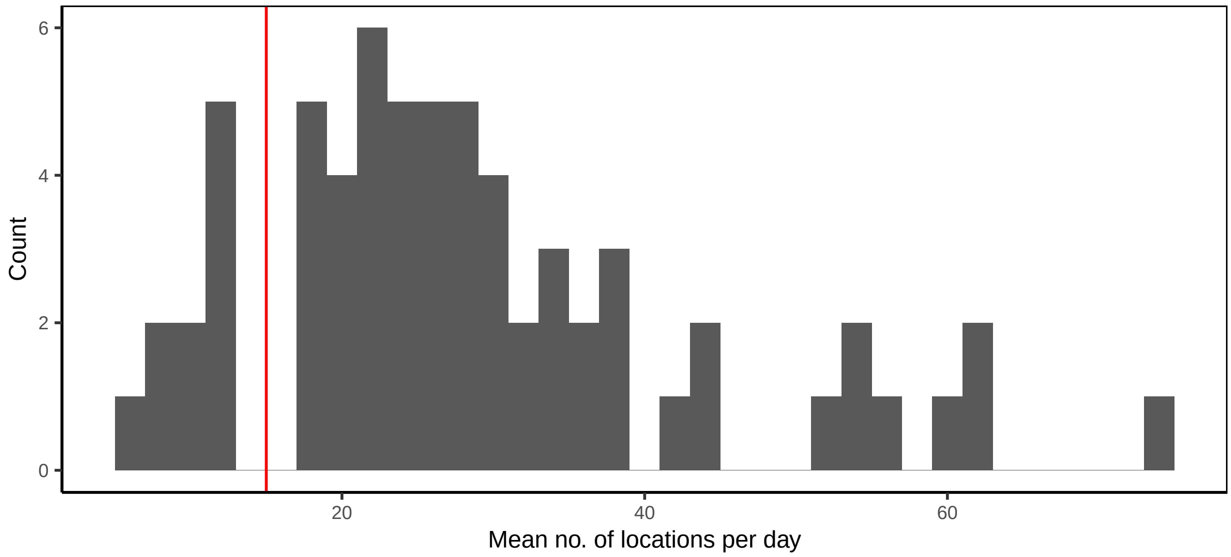

Figure S1: Histogram of the mean number of locations per day for the equipped 65 female SES. The vertical red line shows the threshold of 15 locations per day.

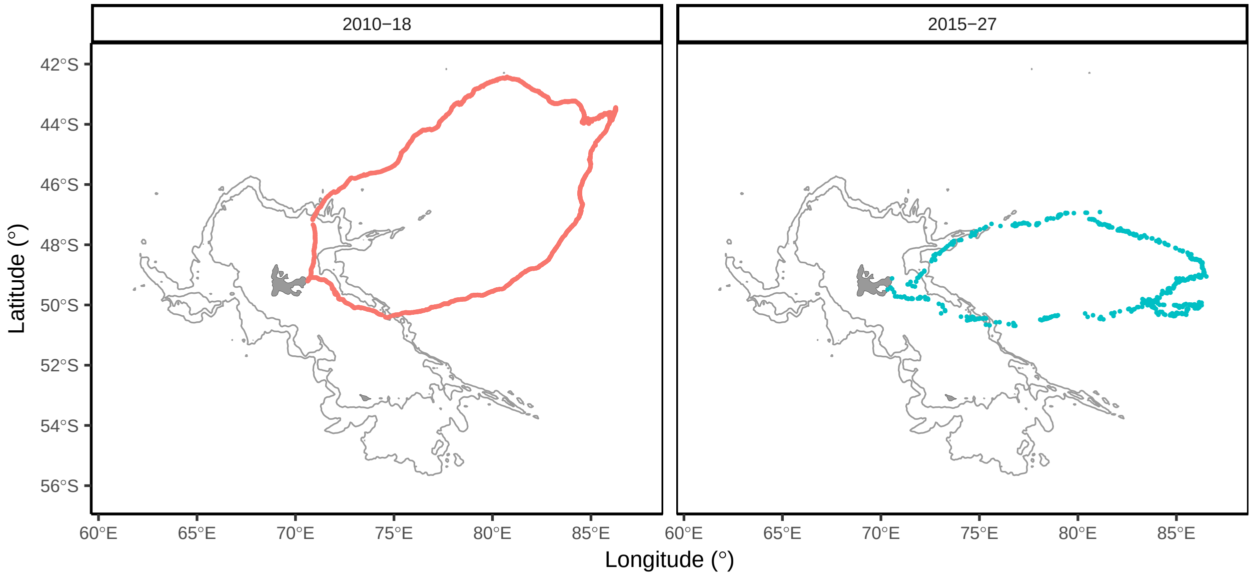

Figure S2: Maps of the locations of two female SES. The seal 2015−27 is an example of the seals that have been excluded from the analysis because of the low sampling rate of recorded locations, whereas the seal 2010−18 is an example of the seals that have been retained in the analysis.

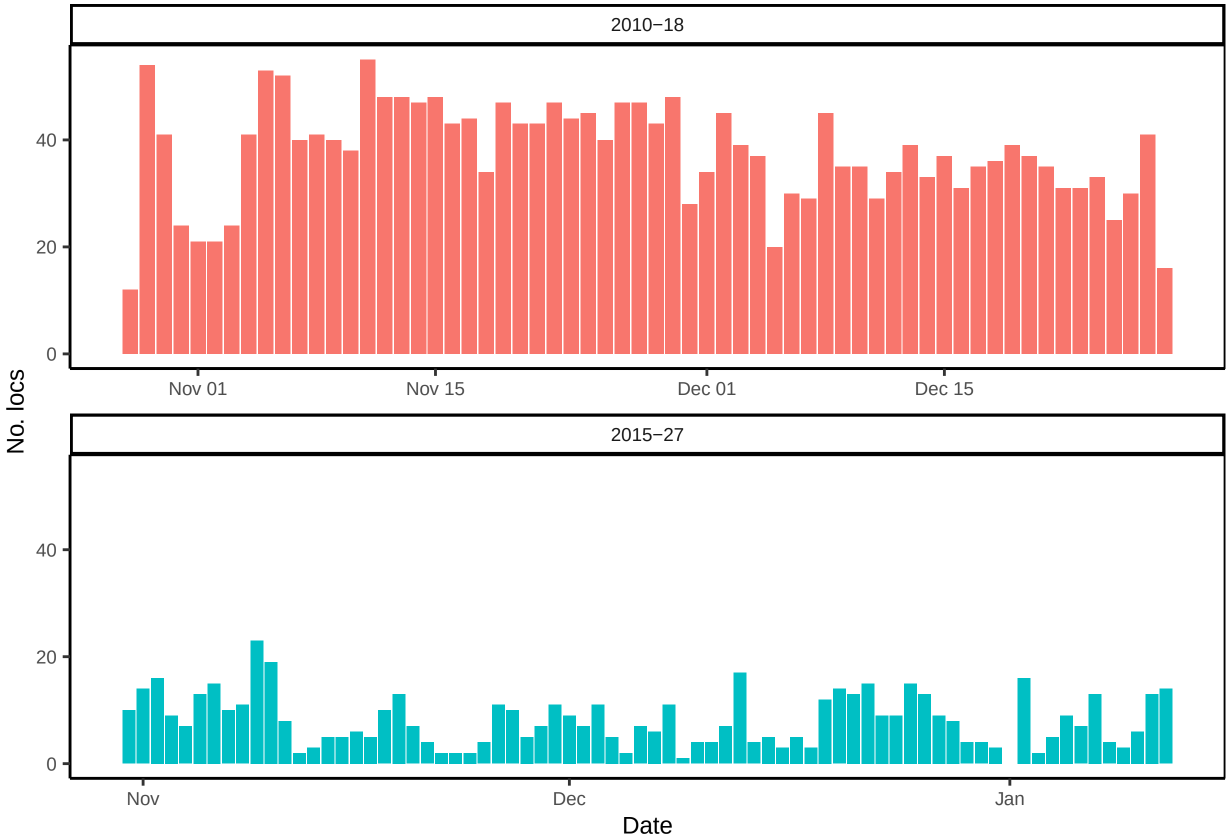

Figure S3: Daily number of locations recorded for two female SES. The seal 2015−27 is an example of the seals that were excluded from the analysis because of the low sampling rate of recorded locations, whereas the seal 2010−18 is an example of the seals that were retained in the analysis.
