# Supplementary material for: Track and dive-based movement metrics do not predict the number of prey encountered by a marine predator
Source: Mov Ecol. 2023 Jan 21;11:3. doi: 10.1186/s40462-022-00361-2 (PMC9862577; doi:10.1186/s40462-022-00361-2)
Supplement: Supplementary file 5 — Additional file 5. Output of all the generalized linear mixed-effect models. [file 40462_2022_361_MOESM5_ESM.pdf]

Additional file 5

Output of all the generalized linear mixed-effect models

Allegue H., Réale D., Picard B., Guinet C. (2022) Track and dive-based movement metrics do not predict the number of prey encountered by a marine predator. *Mov. Ecol.*

Contents

1 GLMM with one of the metrics **1**

2 GLMM with all the dive-based metrics **3**

3 GLMM with all the track-based metrics **5**

4 GLMM with all the metrics **6**

1 GLMM with one of the metrics

Table S1: Output estimates (± se [95% confidence interval]) of all the single-metric GLMM at the scale of dives and days. Dive metrics were calculated from the **high-resolution dive profiles** (i.e., at 1Hz). The GLMM were fitted with a Poisson distribution and a log link function where the number of prey encounter events is specified as the response variable, the focal metric as the fixed effect, and individual seals as random intercepts and slopes. The standard deviation (sd) of the random intercepts and slopes as well as the correlation (Cor) between them are reported.

		Scale of					
		Dives			Days		
Model	Term	Estimate	Confidence interval	Z score	Estimate	Confidence interval	Z score
Descent rate	Intercept	1.91 ± 0.06	[1.79, 2.03]	31.88	6.36 ± 0.08	[6.21, 6.52]	81.21
	Descent rate	0.44 ± 0.02	[0.40, 0.48]	19.72	0.28 ± 0.03	[0.21, 0.34]	8.20
	sd(Intercept)	0.27	[0.20, 0.37]		0.35	[0.26, 0.48]	
	sd(Descent rate)	0.10	[0.07, 0.14]		0.14	[0.10, 0.20]	
	Cor(Intercept, Descent rate)	0.21	[-0.22, 0.55]		0.40	[-0.09, 0.69]	
Ascent rate	Intercept	1.87 ± 0.07	[1.74, 2.00]	28.57	6.35 ± 0.09	[6.17, 6.53]	69.12
	Ascent rate	0.60 ± 0.02	[0.56, 0.64]	29.83	0.42 ± 0.02	[0.37, 0.47]	17.33
	sd(Intercept)	0.30	[0.22, 0.41]		0.42	[0.31, 0.57]	
	sd(Ascent rate)	0.09	[0.07, 0.12]		0.09	[0.06, 0.14]	
	Cor(Intercept, Ascent rate)	-0.11	[-0.48, 0.31]		0.78	[0.27, 0.91]	

Table S1: *(Continued.)*

Model	Term	Estimate	Confidence interval	Z score	Estimate	Confidence interval	Z score
Bottom duration	Intercept	1.91 ± 0.05	[1.80, 2.01]	36.13	6.33 ± 0.07	[6.20, 6.46]	94.74
	Bottom duration	-0.16 ± 0.03	[-0.21, -0.11]	-6.05	-0.02 ± 0.04	[-0.09, 0.06]	-0.42
	sd(Intercept)	0.24	[0.18, 0.33]		0.30	[0.22, 0.41]	
	sd(Bottom duration)	0.12	[0.09, 0.16]		0.15	[0.10, 0.22]	
	Cor(Intercept, Bottom duration)	-0.23	[-0.57, 0.20]		-0.33	[-0.67, 0.19]	
Surface duration	Intercept	1.89 ± 0.05	[1.79, 1.98]	38.13	6.32 ± 0.07	[6.19, 6.45]	92.03
	Surface duration	-0.13 ± 0.04	[-0.21, -0.05]	-3.19	-0.06 ± 0.06	[-0.17, 0.05]	-1.12
	sd(Intercept)	0.23	[0.17, 0.31]		0.30	[0.22, 0.42]	
	sd(Surface duration)	0.18	[0.14, 0.25]		0.23	[0.15, 0.35]	
	Cor(Intercept, Surface duration)	-0.31	[-0.61, 0.13]		-0.05	[-0.50, 0.43]	
Efficiency	Intercept	1.86 ± 0.05	[1.77, 1.95]	41.13	6.31 ± 0.06	[6.19, 6.43]	102.72
	Efficiency	0.16 ± 0.05	[0.07, 0.25]	3.45	0.18 ± 0.05	[0.08, 0.27]	3.60
	sd(Intercept)	0.21	[0.15, 0.28]		0.27	[0.20, 0.38]	
	sd(Efficiency)	0.21	[0.15, 0.28]		0.21	[0.15, 0.30]	
	Cor(Intercept, Efficiency)	0.30	[-0.13, 0.61]		0.40	[-0.08, 0.69]	
Sinuosity	Intercept	1.91 ± 0.06	[1.79, 2.02]	33.14	6.38 ± 0.09	[6.21, 6.55]	74.83
	Sinuosity	0.34 ± 0.03	[0.27, 0.40]	10.28	0.27 ± 0.05	[0.18, 0.36]	5.92
	sd(Intercept)	0.26	[0.19, 0.36]		0.39	[0.28, 0.53]	
	sd(Sinuosity)	0.15	[0.11, 0.20]		0.20	[0.14, 0.28]	
	Cor(Intercept, Sinuosity)	0.14	[-0.28, 0.50]		0.30	[-0.16, 0.62]	
Hunting time	Intercept	1.97 ± 0.09	[1.79, 2.16]	20.82	6.69 ± 0.18	[6.33, 7.04]	36.89
	Hunting time	0.59 ± 0.04	[0.51, 0.68]	13.60	0.65 ± 0.12	[0.42, 0.88]	5.63
	sd(Intercept)	0.43	[0.32, 0.59]		0.82	[0.60, 1.13]	
	sd(Hunting time)	0.20	[0.15, 0.27]		0.52	[0.37, 0.71]	
	Cor(Intercept, Hunting time)	0.74	[0.41, 0.87]		0.87	[0.62, 0.94]	
Horizontal speed	Intercept	1.89 ± 0.05	[1.79, 1.99]	36.68	6.34 ± 0.08	[6.19, 6.49]	83.05
	Horizontal speed	-0.23 ± 0.03	[-0.30, -0.16]	-6.62	-0.28 ± 0.03	[-0.35, -0.21]	-8.03
	sd(Intercept)	0.22	[0.16, 0.31]		0.33	[0.24, 0.45]	
	sd(Horizontal speed)	0.15	[0.11, 0.21]		0.14	[0.10, 0.20]	
	Cor(Intercept, Horizontal speed)	-0.16	[-0.53, 0.28]		-0.39	[-0.69, 0.11]	
Turning angle	Intercept	1.91 ± 0.05	[1.81, 2.01]	37.05	6.40 ± 0.08	[6.24, 6.56]	78.78
	Turning angle	0.01 ± 0.01	[-0.01, 0.03]	1.35	0.28 ± 0.05	[0.18, 0.38]	5.37
	sd(Intercept)	0.22	[0.16, 0.31]		0.35	[0.25, 0.48]	
	sd(Turning angle)	0.04	[0.03, 0.06]		0.20	[0.13, 0.30]	
	Cor(Intercept, Turning angle)	0.01	[-0.43, 0.45]		0.64	[0.06, 0.85]	
FPT	Intercept	1.94 ± 0.05	[1.83, 2.04]	36.29	6.37 ± 0.07	[6.23, 6.52]	85.16
	FPT	0.20 ± 0.02	[0.15, 0.25]	8.35	0.24 ± 0.04	[0.17, 0.31]	6.62
	sd(Intercept)	0.23	[0.17, 0.32]		0.32	[0.23, 0.45]	
	sd(FPT)	0.10	[0.07, 0.14]		0.13	[0.08, 0.21]	
	Cor(Intercept, FPT)	0.67	[0.28, 0.84]		0.80	[0.34, 0.92]	
Move persistence	Intercept	1.89 ± 0.10	[1.70, 2.08]	19.33	6.33 ± 0.09	[6.15, 6.50]	71.07
	Move persistence	-0.33 ± 0.04	[-0.41, -0.24]	-7.54	-0.32 ± 0.05	[-0.41, -0.23]	-6.79
	sd(Intercept)	0.43	[0.31, 0.59]		0.38	[0.27, 0.53]	
	sd(Move persistence)	0.19	[0.13, 0.26]		0.17	[0.12, 0.26]	
	Cor(Intercept, Move persistence)	-0.07	[-0.48, 0.38]		-0.23	[-0.63, 0.32]	

Table S2: Output estimates (± se [95% confidence interval]) of the GLMM including dive-based metrics computed from **low-resolution dive profiles** at the scale of dives and days. The GLMM were fitted with a Poisson distribution and a log link function where the number of prey encounter events is specified as the response variable, the focal metric as the fixed effect, and individual seals as random intercepts and slopes. The standard deviation (sd) of the random intercepts and slopes as well as the correlation (Cor) between them are reported.

		Scale of					
		Dives			Days		
Model	Term	Estimate	Confidence interval	Z score	Estimate	Confidence interval	Z score
Descent rate	Intercept	1.90 ± 0.05	[1.80, 2.01]	35.29	6.36 ± 0.08	[6.21, 6.51]	82.51
	Descent rate	0.29 ± 0.03	[0.24, 0.34]	11.28	0.22 ± 0.04	[0.15, 0.29]	6.14
	sd(Intercept)	0.25	[0.18, 0.33]		0.35	[0.25, 0.47]	
	sd(Descent rate)	0.12	[0.09, 0.16]		0.15	[0.10, 0.22]	
	Cor(Intercept, Descent rate)	-0.25	[-0.58, 0.19]		0.21	[-0.26, 0.58]	
Ascent rate	Intercept	1.85 ± 0.05	[1.74, 1.95]	33.88	6.32 ± 0.08	[6.17, 6.48]	80.04
	Ascent rate	0.32 ± 0.04	[0.25, 0.39]	8.52	0.24 ± 0.04	[0.16, 0.31]	6.06
	sd(Intercept)	0.25	[0.18, 0.34]		0.35	[0.26, 0.49]	
	sd(Ascent rate)	0.17	[0.13, 0.23]		0.16	[0.10, 0.23]	
	Cor(Intercept, Ascent rate)	-0.55	[-0.76, -0.14]		-0.04	[-0.48, 0.43]	
Bottom duration	Intercept	1.89 ± 0.05	[1.78, 1.99]	35.45	6.31 ± 0.07	[6.18, 6.45]	94.26
	Bottom duration	-0.22 ± 0.03	[-0.28, -0.16]	-7.14	-0.01 ± 0.05	[-0.11, 0.08]	-0.23
	sd(Intercept)	0.24	[0.18, 0.33]		0.30	[0.22, 0.41]	
	sd(Bottom duration)	0.14	[0.10, 0.19]		0.20	[0.14, 0.29]	
	Cor(Intercept, Bottom duration)	-0.09	[-0.46, 0.33]		-0.39	[-0.69, 0.10]	
Efficiency	Intercept	1.87 ± 0.05	[1.77, 1.97]	36.11	6.30 ± 0.06	[6.18, 6.43]	97.23
	Efficiency	0.04 ± 0.04	[-0.03, 0.11]	1.05	0.16 ± 0.05	[0.07, 0.25]	3.49
	sd(Intercept)	0.24	[0.17, 0.32]		0.29	[0.21, 0.40]	
	sd(Efficiency)	0.16	[0.12, 0.22]		0.20	[0.14, 0.28]	
	Cor(Intercept, Efficiency)	0.40	[-0.03, 0.67]		0.39	[-0.11, 0.69]	
Hunting time	Intercept	1.90 ± 0.05	[1.81, 1.99]	39.77	6.35 ± 0.07	[6.22, 6.48]	93.42
	Hunting time	0.12 ± 0.02	[0.08, 0.17]	5.35	-0.02 ± 0.04	[-0.11, 0.07]	-0.45
	sd(Intercept)	0.22	[0.16, 0.30]		0.30	[0.22, 0.42]	
	sd(Hunting time)	0.10	[0.08, 0.14]		0.19	[0.13, 0.27]	
	Cor(Intercept, Hunting time)	0.44	[0.00, 0.70]		-0.11	[-0.50, 0.34]	

2 GLMM with all the dive-based metrics

Table S3: Output estimates (± se [95% confidence interval]) of GLMM that includes all the dive-based metrics at the scale of dives and days. Dive metrics were calculated from the high-resolution dive profiles (i.e., at 1Hz). The GLMM were fitted with a Poisson distribution and a log link function where the number of prey encounter events is specified as the response variable, the metrics as fixed effect, and individual seals as random intercepts and slopes. The standard deviation (sd) of the random intercepts and slopes are reported.

	Scale of					
	Dives			Days		
Term	Estimate	Confidence interval	Z score	Estimate	Confidence interval	Z score
Intercept	1.82 ± 0.09	[1.66, 1.99]	21.37	6.35 ± 0.08	[6.20, 6.51]	80.31
Descent rate	0.13 ± 0.02	[0.09, 0.17]	6.24	0.02 ± 0.03	[-0.03, 0.07]	0.68
Ascent rate	0.32 ± 0.02	[0.28, 0.35]	19.08	0.26 ± 0.02	[0.21, 0.30]	11.54
Bottom duration	-0.29 ± 0.03	[-0.35, -0.24]	-11.08	-0.19 ± 0.05	[-0.28, -0.10]	-4.14
Surface duration	0.03 ± 0.01	[0.00, 0.06]	1.98	0.07 ± 0.02	[0.03, 0.12]	3.00
Efficiency	0.25 ± 0.03	[0.19, 0.32]	7.52	0.32 ± 0.04	[0.25, 0.40]	8.09
Sinuosity	0.13 ± 0.02	[0.09, 0.17]	6.74	0.08 ± 0.02	[0.04, 0.13]	3.49
Hunting time	0.38 ± 0.03	[0.32, 0.43]	13.56	0.20 ± 0.05	[0.11, 0.29]	4.28
sd(Intercept)	0.39	[0.29, 0.53]		0.35	[0.26, 0.46]	
sd(Descent rate)	0.09	[0.07, 0.13]		0.10	[0.07, 0.15]	
sd(Ascent rate)	0.07	[0.05, 0.10]		0.08	[0.05, 0.12]	
sd(Bottom duration)	0.12	[0.09, 0.16]		0.19	[0.15, 0.24]	
sd(Surface duration)	0.06	[0.05, 0.09]		0.09	[0.06, 0.13]	
sd(Efficiency)	0.15	[0.11, 0.21]		0.17	[0.13, 0.22]	
sd(Sinuosity)	0.09	[0.06, 0.12]		0.09	[0.06, 0.13]	
sd(Hunting time)	0.13	[0.09, 0.17]		0.19	[0.14, 0.25]	

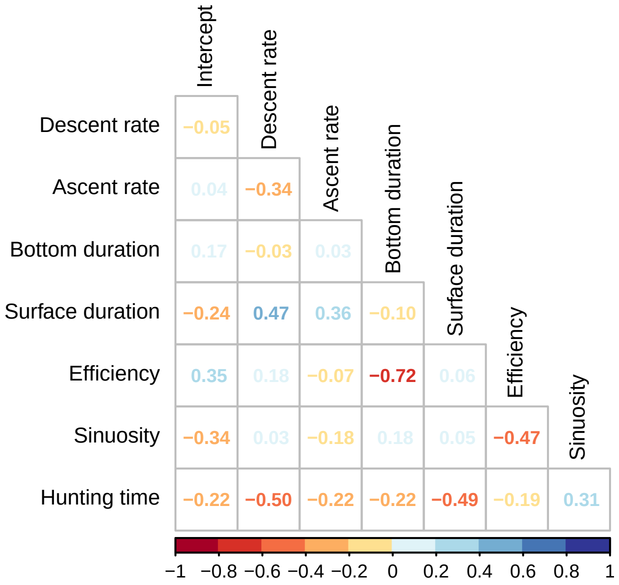

Figure S1: Correlation coefficients between random effects of the GLMM with all the dive-based metrics at the scale of dives.

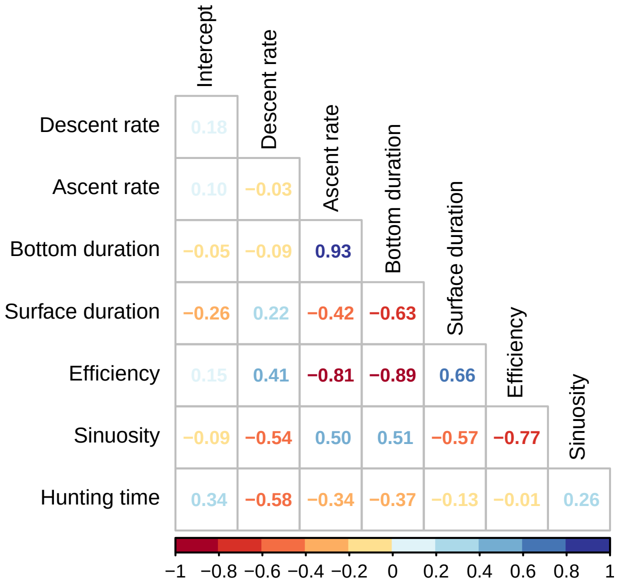

Figure S2: Correlation coefficients between random effects of the GLMM with all the dive-based metrics at the scale of days.

3 GLMM with all the track-based metrics

Table S4: Output estimates (± se [95% confidence interval]) of GLMM that includes all the track-based metrics at the scale of dives and days. The GLMM were fitted with a Poisson distribution and a log link function where the number of prey encounter events is specified as the response variable, the metrics as fixed effect, and individual seals as random intercepts and slopes. The standard deviation (sd) of the random intercepts and slopes are reported.

	Scale of					
	Dives			Days		
Term	Estimate	Confidence interval	Z score	Estimate	Confidence interval	Z score
Intercept	1.85 ± 0.08	[1.69, 2.01]	22.61	6.27 ± 0.07	[6.12, 6.41]	85.15
Horizontal speed	-0.22 ± 0.04	[-0.31, -0.13]	-4.97	-0.33 ± 0.05	[-0.42, -0.23]	-6.68
Turning angle	-0.08 ± 0.01	[-0.10, -0.05]	-6.98	-0.07 ± 0.03	[-0.12, -0.02]	-2.59
FPT	0.02 ± 0.03	[-0.03, 0.07]	0.81	-0.05 ± 0.03	[-0.11, 0.01]	-1.63
Move persistence	-0.19 ± 0.05	[-0.29, -0.08]	-3.53	-0.15 ± 0.06	[-0.27, -0.03]	-2.36
sd(Intercept)	0.36	[0.26, 0.49]		0.30	[0.22, 0.43]	
sd(Horizontal speed)	0.19	[0.14, 0.26]		0.20	[0.15, 0.27]	
sd(Turning angle)	0.04	[0.03, 0.06]		0.06	[0.02, 0.16]	
sd(FPT)	0.11	[0.07, 0.15]		0.08	[0.04, 0.16]	
sd(Move persistence)	0.22	[0.16, 0.31]		0.23	[0.17, 0.33]	

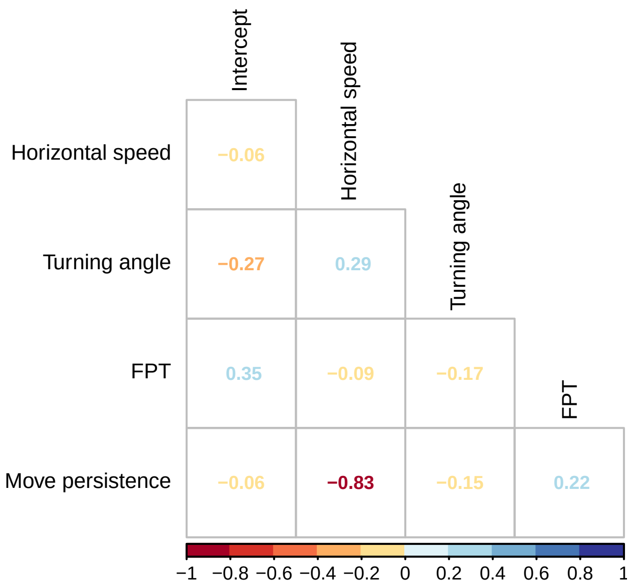

Figure S3: Correlation coefficients between random effects of the GLMM with all the track-based metrics at the scale of dives.

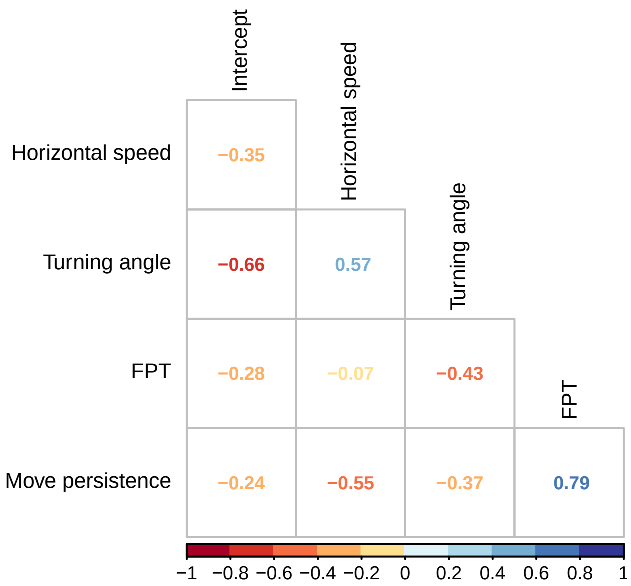

Figure S4: Correlation coefficients between random effects of the GLMM with all the track-based metrics at the scale of days.

4 GLMM with all the metrics

Table S5: Output estimates (± se [95% confidence interval]) of GLMM that includes all the metrics at the scale of dives and days. Dive metrics were calculated from the high-resolution dive profiles (i.e., at 1Hz). The GLMM were fitted with a Poisson distribution and a log link function where the number of prey encounter events is specified as the response variable, the metrics as fixed effect, and individual seals as random intercepts and slopes. The standard deviation (sd) of the random intercepts and slopes are reported.

	Scale of					
	Dives			Days		
Term	Estimate	Confidence interval	Z score	Estimate	Confidence interval	Z score
Intercept	1.76 ± 0.11	[1.54, 1.97]	16.22	6.31 ± 0.10	[6.11, 6.50]	63.01
Descent rate	0.16 ± 0.02	[0.12, 0.21]	7.26	0.03 ± 0.04	[-0.05, 0.11]	0.83
Ascent rate	0.31 ± 0.02	[0.28, 0.35]	18.13	0.24 ± 0.02	[0.19, 0.29]	9.70
Bottom duration	-0.31 ± 0.03	[-0.36, -0.26]	-12.03	-0.20 ± 0.05	[-0.29, -0.11]	-4.47
Surface duration	0.03 ± 0.01	[0.01, 0.06]	2.63	0.06 ± 0.02	[0.02, 0.10]	2.77
Efficiency	0.29 ± 0.03	[0.22, 0.36]	8.53	0.32 ± 0.04	[0.24, 0.41]	7.38
Sinuosity	0.12 ± 0.02	[0.08, 0.16]	5.93	0.07 ± 0.02	[0.03, 0.11]	3.46
Hunting time	0.35 ± 0.03	[0.30, 0.40]	13.94	0.21 ± 0.04	[0.13, 0.29]	5.16
Horizontal speed	-0.04 ± 0.02	[-0.08, -0.00]	-2.16	-0.06 ± 0.02	[-0.10, -0.02]	-2.81
Turning angle	-0.01 ± 0.00	[-0.02, -0.00]	-2.65	-0.02 ± 0.02	[-0.07, 0.03]	-0.88
FPT	0.01 ± 0.02	[-0.04, 0.06]	0.43	0.03 ± 0.02	[-0.02, 0.07]	1.10
Move persistence	0.19 ± 0.13	[-0.06, 0.44]	1.51	0.10 ± 0.05	[-0.01, 0.20]	1.76
sd(Intercept)	0.47	[0.35, 0.64]		0.42	[0.31, 0.56]	
sd(Descent rate)	0.10	[0.07, 0.14]		0.16	[0.13, 0.21]	
sd(Ascent rate)	0.07	[0.05, 0.10]		0.08	[0.06, 0.12]	
sd(Bottom duration)	0.11	[0.08, 0.15]		0.18	[0.14, 0.24]	
sd(Surface duration)	0.05	[0.04, 0.07]		0.06	[0.04, 0.10]	
sd(Efficiency)	0.15	[0.11, 0.20]		0.18	[0.13, 0.23]	
sd(Sinuosity)	0.09	[0.06, 0.12]		0.06	[0.04, 0.10]	
sd(Hunting time)	0.11	[0.08, 0.15]		0.15	[0.11, 0.20]	
sd(Horizontal speed)	0.08	[0.06, 0.11]		0.07	[0.05, 0.10]	
sd(Turning angle)	0.02	[0.01, 0.03]		0.08	[0.05, 0.13]	
sd(FPT)	0.10	[0.07, 0.14]		0.08	[0.05, 0.11]	
sd(Move persistence)	0.55	[0.43, 0.69]		0.22	[0.16, 0.29]	

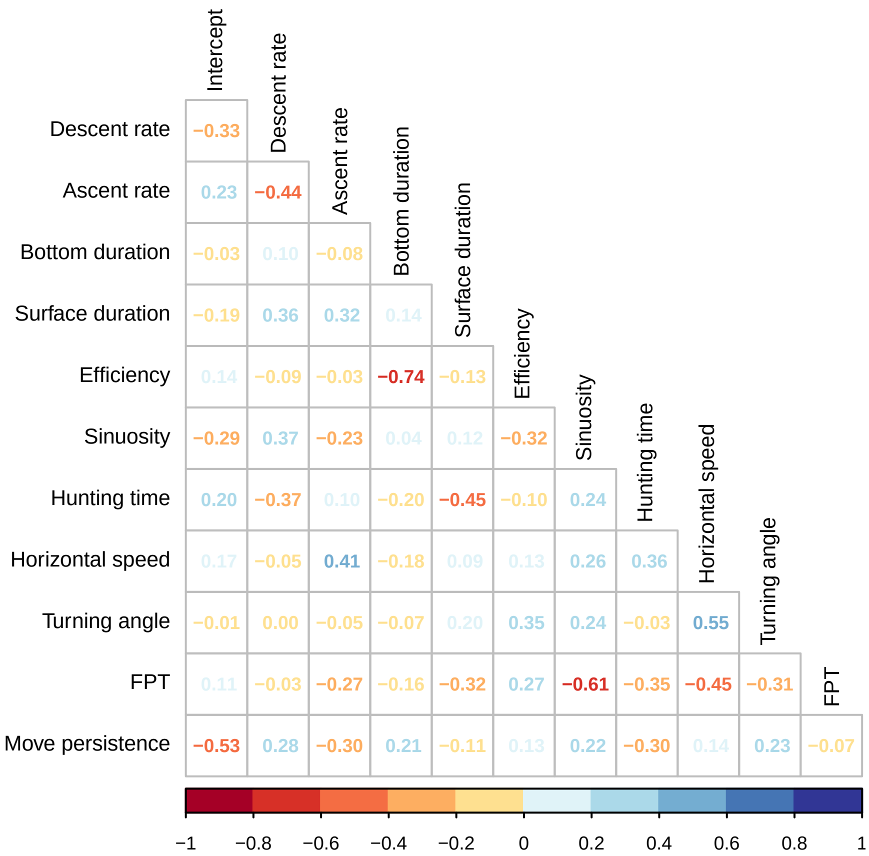

Figure S5: Correlation coefficients between random effects of the GLMM with all the metrics at the scale of dives.

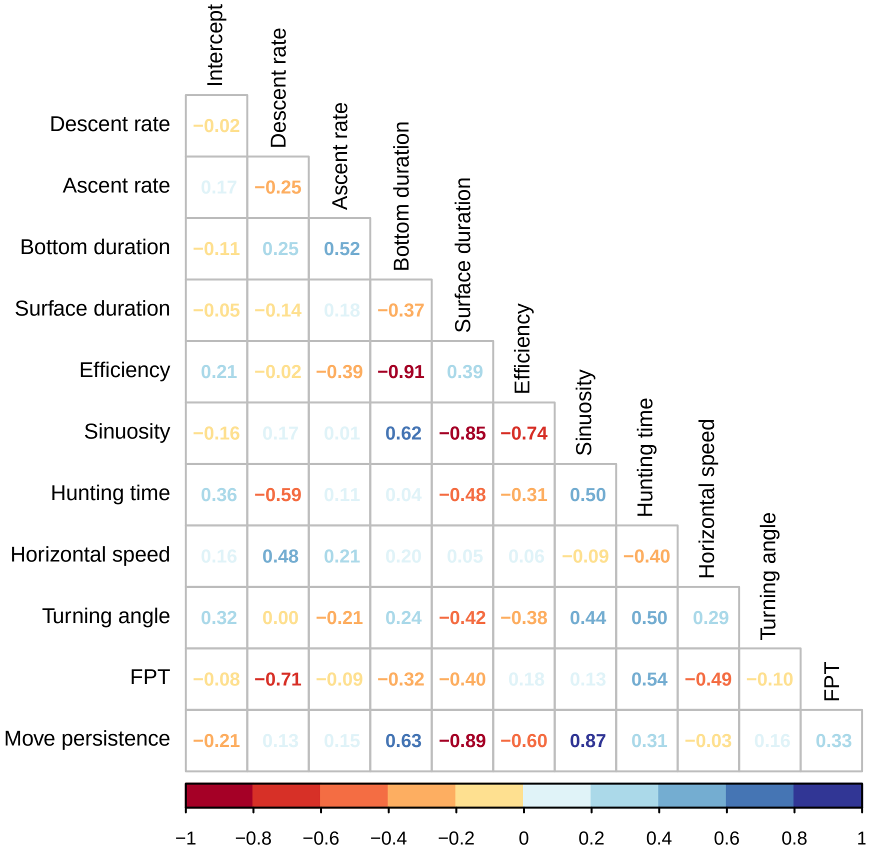

Figure S6: Correlation coefficients between random effects of the GLMM with all the metrics at the scale of days.
